# How high-pressure ices on large ocean worlds can support habitability: The compatibility of amino acids into ice VI and VII

**DOI:** 10.1126/sciadv.aec2045

**Published:** 2026-10-21

**Authors:** Jihua Hao, Can Liu, Feixiang Liu, Chengtao Zhang, Javed Akhter Mondal, Christopher R. Glein, Herve Cardon, Gilles Montagnac, Xiaobin Cao, Isabelle Daniel

**Affiliations:** ^1^ Deep Space Exploration Laboratory/State Key Laboratory of Lithospheric and Environmental Coevolution, University of Science and Technology of China, Hefei 230026, Anhui, China.; ^2^ International Center for Isotope Effects Research, State Key Laboratory of Critical Earth Material Cycling and Mineral Deposits, Frontiers Science Center for Critical Earth Material Cycling, School of Earth Sciences and Engineering, Nanjing University, Nanjing, China.; ^3^ Space Science Division, Space Sector, Southwest Research Institute, San Antonio, TX 78238, USA.; ^4^ Universite Claude Bernard Lyon1, LGL-TPE, UMR 5276, CNRS, Ens de Lyon, Universite Jean Monnet Saint-Etienne, Villeurbanne 69622, France.

## Abstract

Water oceans in large icy moons and exoplanets are isolated from the rocky interior by thick layers of high-pressure ice, which hinder the direct release of nutrients and organics derived from water-rock interaction to the ocean. Possible uptake of these bio-essential compounds into high-pressure ice phases could regulate their availability in the ocean, but this process is poorly constrained. Here, we measured partition coefficients of glycine and alanine into ices VI and VII under elevated temperature-pressure conditions using a diamond-anvil cell. We observed moderate compatibility of glycine and alanine with partition coefficients: *K*_*p*,glycine_ = 0.14 and 0.40 in ices VI and VII, respectively; *K*_*p*,alanine_ = 0.21 and 0.35 in ices VI and VII, respectively. First-principles calculations suggested that amino acids could be incorporated into high-pressure ice’s lattice via cogrowth. Compatibility of amino acids into high-pressure ices would favor their transport in large extraterrestrial oceans, opening an avenue to habitability.

## INTRODUCTION

As liquid water is a prerequisite for the only example of life we know, water-rich planetary bodies hosting liquid water oceans have become preferred targets in the search for planetary habitability (*1*). Apart from Earth, our solar system has several moons and asteroids showing strong evidence of liquid water oceans beneath their icy exteriors, such as Ceres, Europa, Ganymede, Callisto, Enceladus, and Pluto (*2*). Beyond the solar system, there are numerous exoplanets with more than 6000 confirmed detections so far (*3*). Among them, a dozen have been proposed to be able to support liquid water on their surfaces based on their radius and distance to host star (*4*, *5*). Moreover, some exomoons with subsurface oceans are also expected to exist (*6*), although they remain to be found. Multiple international missions were recently launched or will be launched to explore these potentially ocean-bearing worlds, such as JUICE (to Jovian moons; 2023) (*7*), Europa Clipper (to Europa; 2024) (*8*), Dragonfly (to Titan; 2028) (*9*), and Tianwen-IV (to Jovian moons; expected in 2030) (*10*). The James Webb Space Telescope is also operating with a major scientific goal to search for habitable exoplanets and potential biosignatures (*11*).

In view of the existence of ocean worlds, the next critical question for their habitability is the availability of building blocks of life, particularly key biomolecules, e.g., amino acids and nucleotides (*12*). Such species can be inherited during the formation of their star systems and/or supplied later by various sources. For example, planetary building blocks including comets and some meteorites contain substantial levels of organics including amino acids (*13*, *14*), which may partially survive delivery from space and impact heating on icy moons and exoplanets. Moreover, abiotic synthesis of organics, including amino acids, by the reduction of CO/CO_2_ could occur during aqueous alteration of Fe(II)-rich rocks (*15*–*17*), which provides catalytic minerals and produces H_2_ (*18*). On atmosphere-bearing icy moons (e.g., Titan) or exoplanets, abiotic synthesis of organics could also occur in the reducing atmospheres via radiation (*19*) or lightning (*20*). Although amino acids have not yet been detected on any contemporary extraterrestrial ocean worlds, small organic compounds with -NH_2_ and -COOH functional groups have been found in Enceladus’ plume (*21*) and Titan’s atmosphere (*22*).

Beyond these possible sources, the accumulation of building blocks of life in surface or subsurface liquid water oceans would also depend on the internal structure of the planetary bodies. For example, there is expected to exist a layer (or layers) of high-pressure ice(s) between the liquid water layers and the deeper silicate interior in some large icy moons (e.g., Ganymede and Callisto) and super-Earths (or sub-Neptunes) (*23*), potentially hindering the exchange of materials between the ocean and silicates (*24*). However, recent geodynamic models suggest that some thermal plumes of deeply originated meltwater can upwell through two-phase convection involving melts in high-pressure ices, helping transport solutes to the overlying ocean (*25*, *26*). During the upwelling, these meltwater plumes would continuously interact with surrounding high-pressure ices and some may even undergo cycles of freezing and re-melting (*26*). Thus, the presence of high-pressure ice(s) and its interaction with aqueous solutes may largely affect the accumulation of bio-essential species in the oceans of large icy moons and exoplanets.

Equilibrium freezing of ice is generally believed to expel solutes with very low partition coefficients (*27*), e.g., the ejection of NaCl by ice Ih under ambient conditions on Earth. However, high-pressure experiments have revealed substantial incorporation of inorganic salts, such as NH_4_F, NaCl, KCl, LiCl, LiBr, and RbI, into high-pressure ices (VI, VII, or X) (*28*–*37*). This partitioning was proposed to strongly affect the transport of salts to extraterrestrial oceans (*34*, *38*). Unlike inorganic species, there has been little work demonstrating the partitioning of organic molecules into high-pressure ices. One exception is the study of Frank *et al.* (*29*) who reported substantial incorporation of methanol (CH_3_OH) into ice VII at high P. However, the experiment of Frank *et al.* (*29*) was conducted in a totally frozen state without any estimation of the partition coefficient, and in this case, CH_3_OH was likely forced into ice VII.

In the present study, we conducted equilibrium freezing experiments with amino acid (glycine and alanine) solutions at elevated *T* (up to 200°C) and *P* (up to 3.3 gigapascal or GPa) using a diamond-anvil cell (DAC). Among the proteinogenic amino acids, glycine and alanine are the two simplest, and they are also abundant species in prebiotic systems such as carbonaceous chondrites (*39*). Our experiments were run at temperatures and pressures close to the melting curves of ices VI and VII (fig. S1), which simulate *T*-*P* conditions at the interface of liquid water and icy mantles in some large ocean worlds (*5*, *25*). To evaluate the relative concentrations of amino acids (*c*_AA_) in two phases, we measured in situ Raman spectra of the high-pressure ices and its coexisting aqueous solutions near the phase boundary using the same instrumental settings. Then, we carefully selected the data based on the characteristic peaks of high-pressure ice with no or minimal contribution of fluid, and estimated the partition coefficients (*K*_*p*_ = $\frac{C_{\text{AA},\text{ice}}}{C_{\text{AA},\text{liquid}}}$) (Materials and Methods), defined as the equilibrium ratio of amino acid concentration in the high-pressure ice phase to that in the coexisting aqueous phase. We further conducted first-principles calculations to reveal the molecular interactions between amino acids and water molecules during the crystallization of ices VI and VII.

## RESULTS

### General observations of high-pressure experiments

In our experiments, we did not observe any precipitation or polymerization of glycine or alanine even at a large extent (>80%) of freezing during our slow thawing-freezing cycles (e.g., Fig. 1A and figs. S3 and S4). Any precipitate of glycine or alanine zwitterions would have been easily observed and detected due to the sensitivity of their Raman spectra to their conformation (*40*). Thus, the lack of such precipitates qualitatively indicates that a substantial proportion of amino acids should be incorporated into ices VI and VII during the equilibrium freezing in this study.

**Fig. 1.**
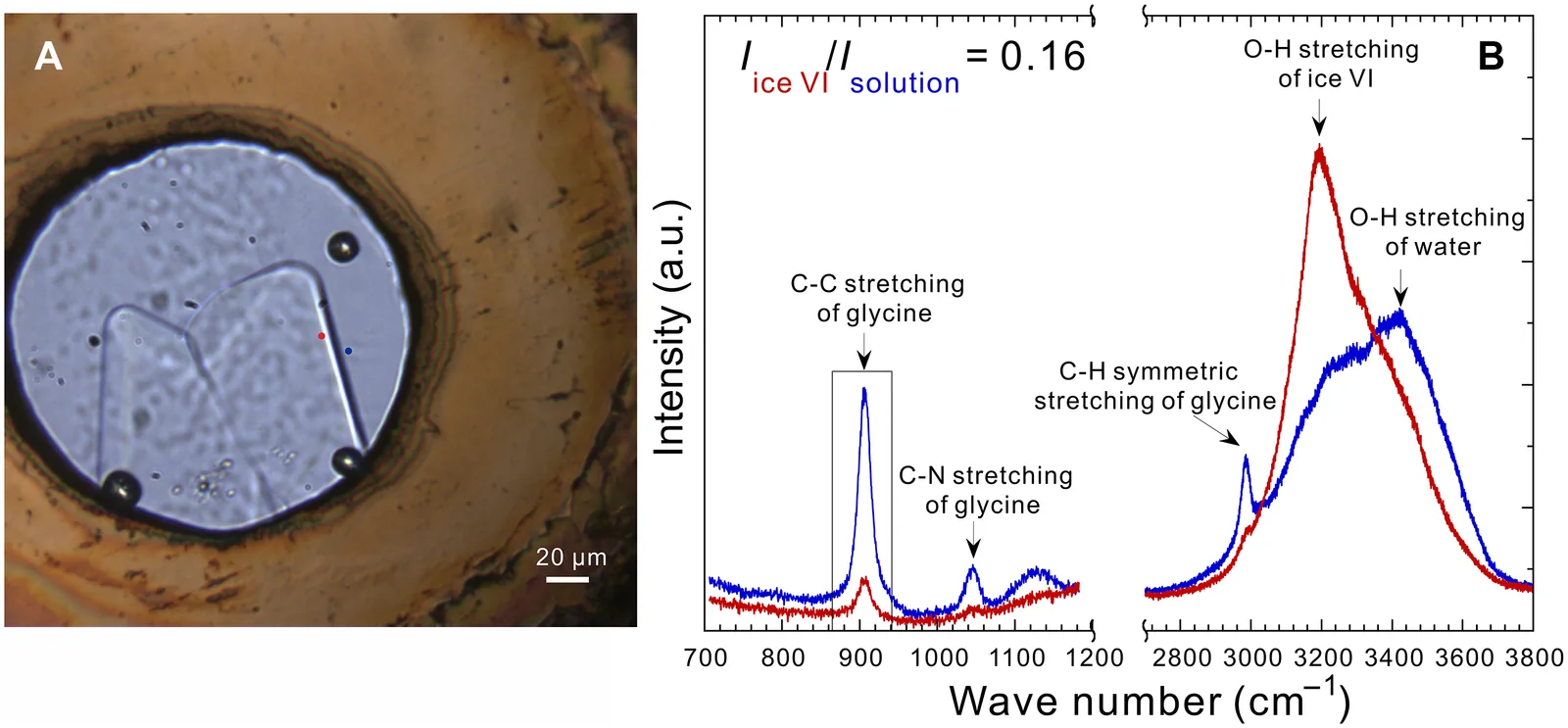
Partitioning of glycine between ice VI and water (1 mol kg^−1^). (**A**) Image of the sample in the diamond anvil cell at 30°C and 0.79 GPa; transmitted light; scale bar, 20 μm. Blue and red dots show the locations where Raman spectra were typically measured in the fluid and ice VI phase, respectively. (**B**) Corresponding Raman spectra with same color code used in (A). The box centered at 900 cm^−1^ shows the characteristic peak of glycine, whose area was used for the calculation of the partition coefficient. See assignment in the main text.

### Partitioning of glycine and alanine between ice VI and aqueous solution

For the estimation of partition coefficients of amino acids, we carefully selected Raman spectra of ice VI (and its nearby fluid) based on the shape and relative intensity of O─H symmetric stretching peaks at high wave numbers (3000 to 3800 cm^−1^). Figure 1B shows one example spectrum displaying the characteristic signal of ice VI without any contribution of liquid water. Using these selected pairs of spectra, we estimated partition coefficients ${Kp}_{\text{Gly}}^{\text{VI}-l}$ and ${Kp}_{\text{Ala}}^{\text{VI}-l}$ between ice VI and coexisting fluid using Eq. 2 in Materials and Methods as a function of temperature and pressure along the melting curve (Fig. 2). Here, each displayed *K*_*p*_ value is the average of multiple analyses at different locations along the ice-fluid boundary under the same *T*-*P* conditions. The overall uncertainty of the estimated partition coefficients represents the SD of those analyses (tables S1 and S2), while the variability of an individual analysis at one location never exceeds 12%.

**Fig. 2.**
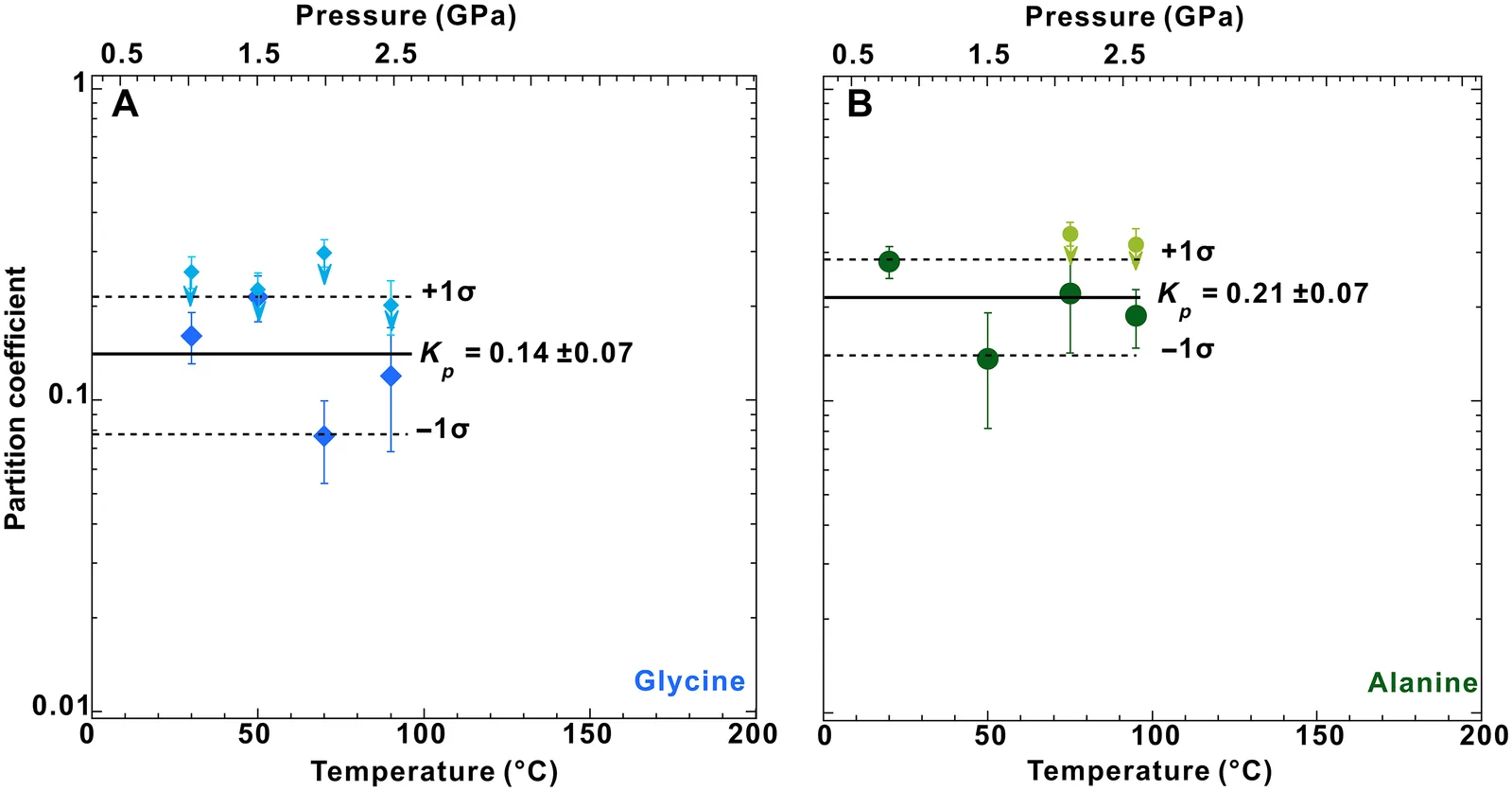
Partition coefficients of glycine and alanine between liquid water and ice VI at temperatures from 20° to 100°C and pressures from 0.8 to 2.6 GPa. (**A**) Glycine (blue diamonds). (**B**) Alanine (green filled circles). Dark-colored symbols show experimental data devoid of fluid contribution (see tables S1 and S2 for the raw data). Error bars indicate the associated uncertainty evaluated from several analyses in different locations where fluid and ice equilibrated, at the same *T*-*P*. Light-colored symbols correspond to experimental data where a minor contribution from fluid was suspected from the subtle change of the O─H stretching band. The light-colored symbols thus represent upper limit values of *K*_*p*_ as indicated by downward arrows. The mean *K*_*p*_ values are shown as a black horizontal line. Dashed lines correspond to ±1 σ standard error.

Under the *T*-*P* conditions investigated here, ${Kp}_{\text{Gly}}^{\text{VI}-l}$ is constant (0.14 ± 0.07; Fig. 2A). There is no detectable dependence of the partition coefficient on either temperature or pressure, potentially due to the narrow stability melting *T*-*P* conditions of ice VI, between 0°C–0.6 GPa (triple point of ice V–ice VI–liquid) and 80°C–2.2 GPa (triple point of ice VI–ice VII–liquid) (fig. S1), and a very small Clapeyron slope of 0.024 GPa °C^−1^ [see Journaux *et al.* (*41*) for specific values]. In addition, we find that the estimated partition coefficients of alanine between ice VI and the fluid (${Kp}_{\text{Ala}}^{\text{VI}-l}$) are close to that of glycine with an average value of 0.21 ± 0.07 under the investigated *T*-*P* conditions (Fig. 2B). One example of the Raman spectrum of ice VI equilibrated with an aqueous alanine solution is displayed in fig. S3B, which again highlights the sharp signal of ice VI at high wave numbers, without the contribution of liquid water. Similar to glycine, we did not observe a detectable dependence of the estimated partition coefficient of alanine on temperature or pressure, either.

### Partitioning of glycine and alanine between ice VII and aqueous solution

Spectra of ice VII and its coexisting aqueous solution were measured at temperatures between 25° and 170°C and pressures between 1.2 and 3.4 GPa. We applied the same criteria as mentioned above to select spectra of ice VII devoid of fluid contribution for the estimation of partition coefficients (see fig. S3, A and C, for examples). Figure 3 shows the estimated partition coefficients ${Kp}_{\text{Gly}}^{\text{VII}-l}$ and ${Kp}_{\text{Ala}}^{\text{VII}-l}$ between ice VII and coexisting fluid as a function of temperature and pressure. Glycine and alanine are both slightly less incompatible (more incorporated) in ice VII than in ice VI, with ${Kp}_{\text{Gly}}^{\text{VII}-l}$ = 0.40 ± 0.15 and ${Kp}_{\text{Ala}}^{\text{VII}-l}$ = 0.35 ± 0.15. Similar to the case of ice VI, there is no noticeable dependence of the estimated partition coefficient on temperature or pressure.

**Fig. 3.**
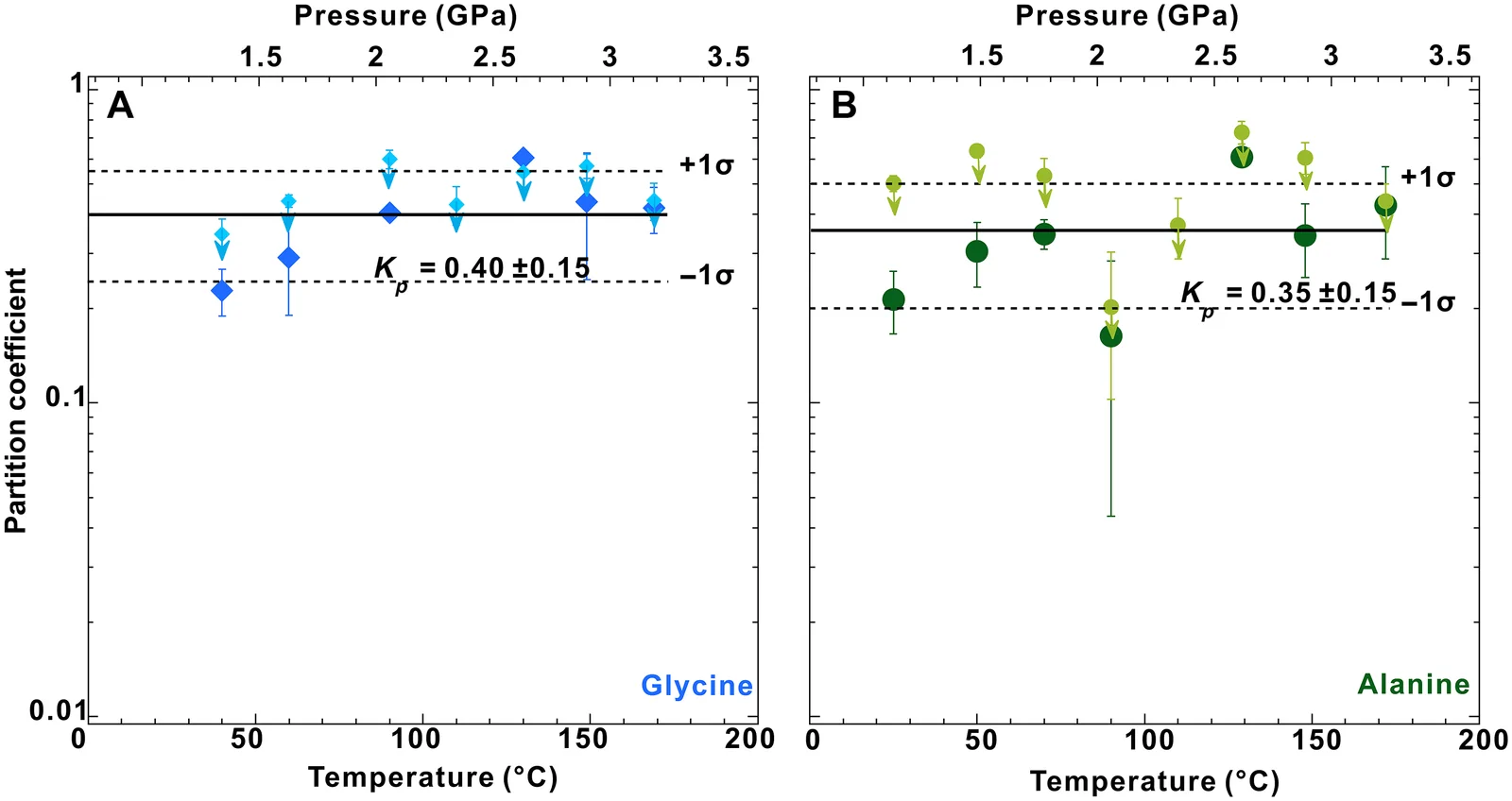
Partition coefficients of glycine and alanine between liquid water and ice VII at temperatures between 25° and 170°C and pressures from 1.2 to 3.4 GPa. (**A**) Glycine (blue diamonds). (**B**) Alanine (green filled circles). Note that this figure uses the same color code as that in Fig. 2. The raw data are provided in tables S1 and S2.

## DISCUSSION

Here, we observed substantial incorporation of amino acids into high-pressure ices (VI and VII). To elucidate the mechanisms and feasibility of amino acid partitioning into these ice structures, we performed first-principle molecular dynamics (MD) simulations and static structural optimizations (see Materials and Methods). Our results show that glycine and alanine form stable coordination environments through hydrogen bonds between their amino, hydroxyl, and carbonyl groups and surrounding water molecules (Fig. 4 and fig. S8), while alkyl groups remain nonbonding. Structural optimizations support direct integration into the extended hydrogen-bond network rather than occupancy of clathrate-like cages. These findings are consistent with prior low-pressure simulation studies (*42*–*44*).

**Fig. 4.**
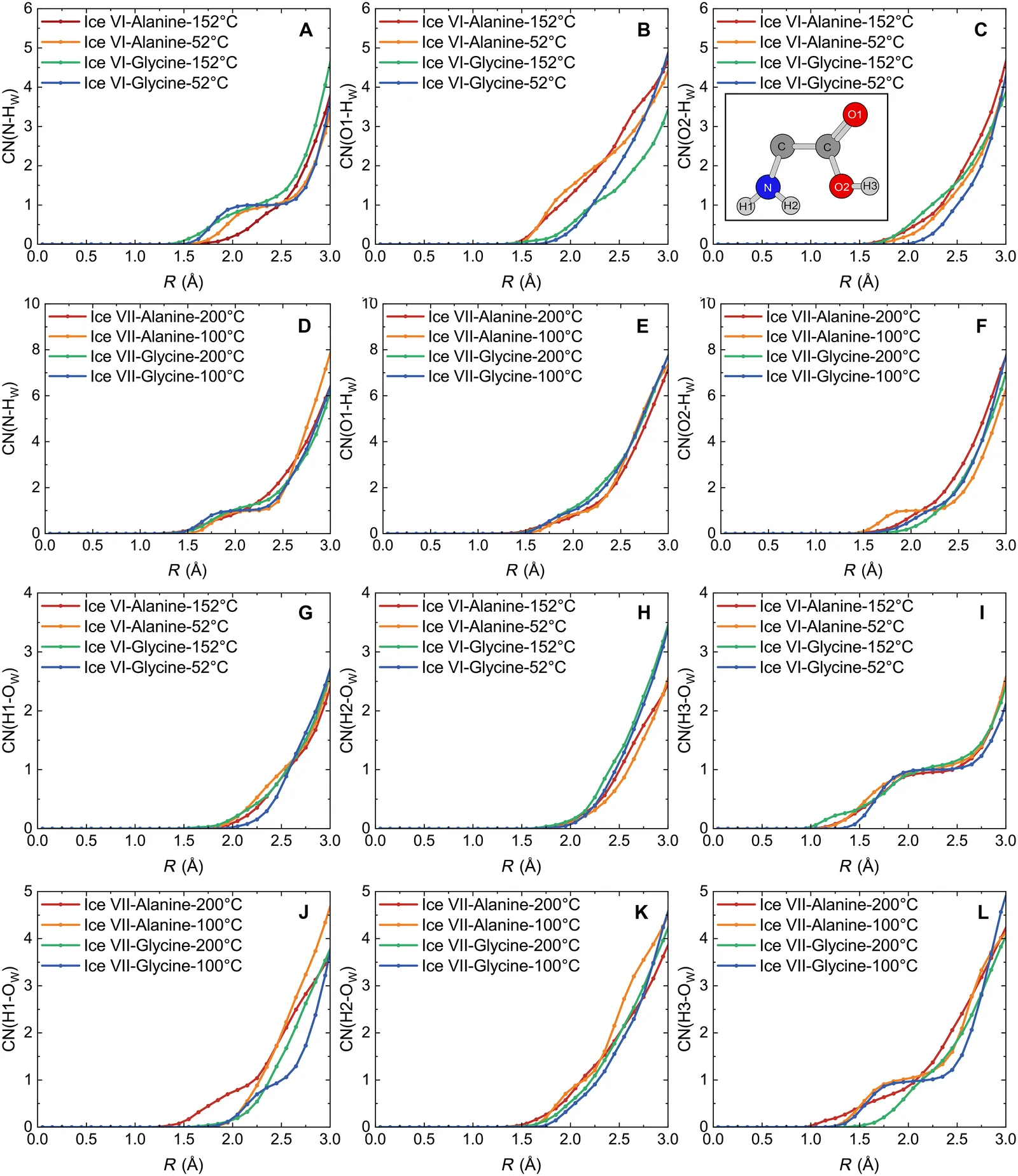
Coordination number functions, *CN*(*R*), between amino acids and water molecules at 52°, 100°, 152°, and 200°C. (**A** to **F**) *CN*(*R*) between amino acids and water H atoms (H_W_) in ice VI (A to C) and ice VII (D to F). (**G** to **L**) *CN*(*R*) between amino acids and water O atoms (O_W_) in ice VI (G to I) and ice VII (J to L). *CN*(*R*) values are integrated from 0 to 3 Å. O1 denotes carbonyl oxygen atoms; O2, hydroxyl oxygen atoms; H1 and H2, amino-group hydrogen atoms; and H3, hydroxyl hydrogen atoms. Red and orange lines correspond to alanine in ice VI/VII from NVT simulations at 152°/200°C and 52°/100°C, respectively; green and blue lines correspond to glycine in ice VI/VII at the same temperatures. At *R* = 2.5 Å, *CN*(*R*) is ∼6 for ice VI (both amino acids, 52° and 152°C) and ∼6 to 7 for ice VII (both amino acids, 100° and 200°C).

Coordination number functions, *CN*(*R*; *R* is the radial distance), can quantitatively characterize these hydrogen-bonding interactions. At *R* = 2.5 Å (*45*), ice VII exhibits higher coordination numbers (∼6 to 7) than ice VI (∼6) regardless of amino acid type (Fig. 4) between glycine and alanine. This arises from distinctive structural features in ice VII: In alanine-bearing systems, N─H_W_ bonds are shorter and a pronounced *CN*(*R*) plateau appears at 1.75 to 2.0 Å for O2─H_W_ interactions, while both alanine- and glycine-bearing ice VII have shorter H3─O_W_ bonds than in ice VI. These features indicate that ice VII supports a denser, more interconnected hydrogen-bond network (more similar to liquid water), likely explaining the observed higher partition coefficients of amino acids (Figs. 2 and 3). Notably, the *CN* of amino acids in ices are close to those in liquid water (*46*, *47*), suggesting similar local hydration environments of amino acids in liquid water and high-pressure ices.

In principle, nuclear quantum effects (NQEs) can influence hydrogen-bond geometry and network ordering in liquid water and solid ice, especially at low temperatures and high pressures (*48*–*53*). Under the conditions examined here (<4 GPa, >27°C), their impact on structural parameters is expected to be minimal (<1%) (*49*, *50*, *53*, *54*). Prior studies show that NQEs primarily broaden the first O─H radial distribution peak and slightly shift the second, with negligible effect on *CN*(*R*) at high pressures (*48*, *52*). Therefore, neglecting NQEs is unlikely to alter our conclusion that amino acids bind directly to the water lattice in ices VI and VII.

Additional evidence of the partitioning comes from the shape of the O─H symmetric stretching band of H_2_O ices measured between 2800 and 3800 cm^−1^ (Fig. 1B and fig. S3), which was found to be broader and relatively more symmetric, with depressed intensity at high wave numbers, compared with that of pure H_2_O ices VI and VII under the same *T*-*P* conditions. These signatures likely reflect the large distortion of hydrogen bonds by the incorporated amino acids (and NaCl, especially for ice VII) (*36*, *55*), similar to the phenomena observed in eutectic systems with methanol at high *T*-*P* (*41*, *56*) and amino acids under ambient conditions (*57*). Compared with Raman spectroscopy, in situ neutron diffraction [e.g., Klotz *et al.* (*37*)] may provide more detailed observational evidence but is beyond the scope of current study.

Apart from the partitioning, surface adsorption of aqueous glycine and alanine onto the ices might have contributed to the observed inclusion (*58*, *59*). However, in the present eutectic system, the amounts of amino acids potentially adsorbed onto the surface of a large single ice crystal would be negligible compared to high contents of amino acids measured in ice VI and VII. Moreover, our Raman spectroscopic measurements of ice samples were all conducted a few micrometers (out of 200 μm in diameter) away from the boundary of ice and fluid where surface adsorption would mainly occur (Fig. 2 and figs. S3 and S4). Therefore, our measurements should represent the signals of amino acids within high-pressure ices rather than adsorbed on their surface.

Under elevated pressures and relatively low temperatures, clathrate hydrates can, in principle, form, and, in some systems, are known to host appreciable concentrations of small organic molecules. However, clathrate formation is unlikely to be relevant to our experiments for several reasons. First, Raman spectra of organic clathrates typically display distinctive, intense bands at 2900 to 2950 cm^−1^ assigned to symmetric C─H symmetric stretching vibrations (*60*, *61*), whereas our spectra are dominated by broad bands at 3000 to 3600 cm^−1^, corresponding to the O─H stretching vibrations of water molecules in ice (Fig. 1B and fig. S3). Second, amino acids are generally discussed in the hydrate literature as kinetic promoters or inhibitors of CO_2_ and CH_4_ hydrate formation, rather than as canonical clathrate guests (*62*, *63*). More fundamentally, clathrate hydrates are water-rich inclusion compounds that require suitable cage-forming guest species, whereas our experiments involve strongly hydrated amino acids at a low amino acid:H_2_O molar ratio (∼1:55) and were specifically designed to investigate partitioning between aqueous fluid and high-pressure ice, rather than guest enclathration (*64*, *65*). In addition, our experiments began by melting most of the ice sample and then slowly growing a small, typically euhedral crystal at *T*-*P* conditions very close to the melting curves of ices VI and VII (fig. S1), but well outside the stability field of clathrate hydrates (*66*). Last, chloride further disfavors hydrate formation, as NaCl is a well-established thermodynamic inhibitor of clathrates (*61*, *66*–*68*). Together, the formation of clathrates of glycine or alanine is highly unlikely in our system.

At elevated temperatures, amino acids may also undergo either polymerization to form di-peptides (*69*) or degradation to produce simpler carboxylic acids along with CO_2_ and NH_3_ (*70*). However, we did not observe the formation of gas bubbles during our experiment. Moreover, there are no clear variation in the Raman spectral intensity of amino acids and additional characteristic peaks of polymerization/degradation products. At *T* < 170°C (highest temperature used in the experiment), the half-life times of glycine [>118 days; based on the reaction kinetics data compiled by Truong *et al.* (*71*)] and alanine (>17.8 days) are expected to be much longer than the experiment duration (<2 days). Previous studies also suggest that the elevation of pressure (to GPa) would tend to stabilize glycine and alanine (*72*). Given these lines of evidence, there should be little, if any, transformation of glycine or alanine in our system.

The present results provide a quantitative or semiquantitative evaluation of the equilibrium partitioning behavior of key biomolecules into high-pressure ices VI and VII. A previous study reported the incorporation of 5 mol % (or 2.8 mol kg^−1^ H_2_O) methanol into ice VII, leading to distortion of hydrogen bonds and expansion of the molecular volume of ice VII (*29*). However, in their x-ray diffraction analyses, the *K*_*p*_ of methanol was not measured as the experiment was not performed at eutectic conditions, and the powderized ice VII did not allow the refinement of the structure of ice VII “doped” with methanol in their experiment. Our estimated values of *K*_*p*_ of amino acids are close to the reported *K*_*p*_ of RbI in ice VII (∼0.12 ± 0.05) but higher than *K*_*p*_ values of RbI in ice VI (5.0 ± 2.0 × 10^−3^) (*36*). Although we used NaCl in the system, these ionic species salts do not show any intrinsic Raman signals and, thus, remain undetected here. Nevertheless, NaCl may have similar or higher *K*_*p*_ values, due to its smaller radius (*36*), consistent with the lack of NaCl precipitation even at a large extent of freezing. These results also echo another study showing that ice VII could integrate several moles of NH_4_F and LiCl per kilogram of water following a specific thermodynamic *T*-*P* path (*35*, *37*).

### Implications for the habitability of large ocean worlds

In this study, we investigated the eutectic system of glycine and alanine aqueous solutions at elevated *T*-*P* and observed that substantial amounts of glycine and alanine can be incorporated into ices VI and VII. These observations could have important implications for the availability of amino acids in the oceans of large moons and exoplanets. In planetary systems, amino acids can be synthesized during water-rock interactions under hydrothermal conditions. For large ocean worlds, hydrothermal activities may occur at the interface between a high-pressure ice layer and a silicate mantle/core, where melting of the ice can be induced by radiogenic or tidal heating (Fig. 5) (*73*). There is also evidence suggesting substantial amounts of organic matter in rocky cores, probably a reservoir inherited from the accretion of comets and chondrites (*74*, *75*). Under these circumstances, hydrothermal alteration of these carbonaceous materials may produce amino acids with minerals acting as possible catalysts (*16*). Under favorable geochemical conditions, amino acids could also be synthesized via the reduction of inorganic C (e.g., CO_2_ and CO) by H_2_ released from serpentinization reactions of mafic/ultramafic rocks (*17*, *76*–*78*). Given long-lived radiogenic and tidal heating, alteration of carbonaceous matter or silicates at the ice-mantle boundary may synthesize substantial amounts of amino acids.

**Fig. 5.**
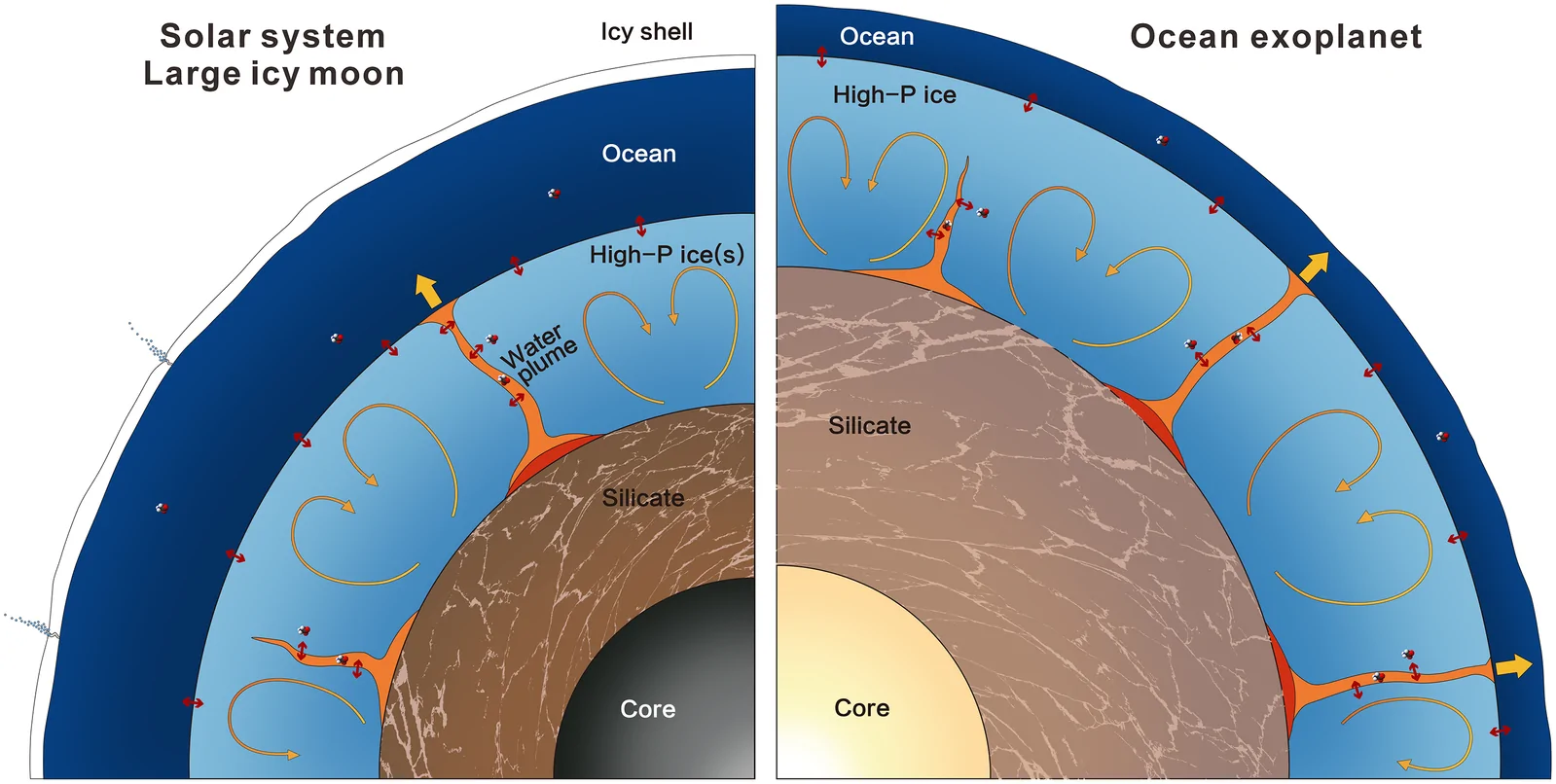
A schematic illustration of potential material-transport pathways through high-pressure ice layer(s) in large icy moons (left) or ocean exoplanets (right). In these large extraterrestrial oceans, amino acids and other dissolved solutes may be depleted through partitioning into the high-pressure ice mantle. Nevertheless, these building blocks of life could be transported from the silicate-ice boundary, where they are synthesized or released, through heat-pipe plumes or solid-state convection within the high-pressure ice mantle. Red arrows indicate the partitioning of amino acids between the aqueous solution and high-pressure ice phase(s).

Recent geodynamic studies suggest that thick high-pressure ice layers may not behave as purely stagnant barriers, and that transport across them can occur in several distinct regimes (Fig. 5) (*25*, *26*, *79*–*82*). In particular, melt generated at the ice-silicate boundary may be buoyant relative to surrounding high-pressure ice and can be transported upward within localized hot upwellings (*25*, *26*). Yet, during ascent, the melt may interact continuously with surrounding high-pressure ice and undergo repeated freezing-melting cycles, leading to variable degrees of chemical exchange and, in some cases, indirect or episodic connection between the deep interior and the ocean (*26*, *80*, *81*). For thicker high-pressure ice layers, the transport may be dominated by plume-focused or heat-pipe–like upwelling (*26*, *79*).

In such a two-phase transport framework, solutes, including salts and amino acids, dissolved in ascending melts could progressively partition into surrounding ice VI or VII, but they could also be reincorporated into liquid phases during subsequent remelting (Fig. 5) (*36*). Thus, high-pressure ice layers may act not only as partial barriers, but also as transient reservoirs and redistribution media for solutes. Recent salt-partitioning experiments show that solutes can be redistributed between aqueous fluids and high-pressure ice phases (*36*). By analogy, if endogenous amino acids are incorporated into high-pressure ice through equilibrium partitioning or trapped within fluid inclusions near the ice-silicate boundary, they may subsequently be transported upward through plume-assisted or convective processes and released again during remelting, although the efficiency and continuity of such transfer are likely to depend strongly on the prevailing geodynamic regime. The resulting aqueous microenvironments may generate chemical gradients potentially favorable for biological activity, provided that the high-pressure conditions do not preclude life per se (*83*). Future work should therefore couple experimentally constrained partition coefficients with geophysical models that explicitly account for plume-focused melt transport, intermittent connectivity, and remelting-driven redistribution in large ocean worlds.

Apart from interior geochemical sources of amino acids, there are also several possible sources at the top of the ocean. For example, on some large ocean worlds having a thick atmosphere [like Titan or Hycean worlds (*84*)], abiotic synthesis of amino acids can be achieved by irradiation of high-energy light (e.g., UV, gamma-ray, and x-ray) or charged particles or lightning on reducing gases, such as H_2_, CH_4_, CO, or NH_3_ (*19*, *20*). In addition, amino acids could also be supplied by impacts of comets and asteroids onto large ocean worlds. For example, glycine (and, in fewer cases, alanine) was detected in cometary bodies in our solar system (*85*–*88*) and recently beyond (*89*). Similar to comets, asteroids that originated in the outer solar system also contain low concentrations of glycine (and less alanine) (*90*, *91*). In addition to the direct delivery, previous studies also proposed impact-induced synthesis of amino acids on planetary surfaces (*92*, *93*). These amino acids, after their transport to the ocean, could partially partition into high-pressure ice phases in the planetary interior as well. Overall, the availability of amino acids as key building blocks of life in large ocean words relies not only on their potential sources but also on how they distribute among different reservoirs, particularly the partitioning into high-pressure ices in the present case.

In large extraterrestrial ocean worlds, the massive overburden of water produces high-pressure ice(s) that impede the direct supply of building blocks of life synthesized at the ice-silicate boundary to the overlying liquid-water ocean. Here, we combined high *T*-*P* experiments and first-principles calculations to investigate the compatibility of glycine and alanine with high-pressure ices (VI and VII) from liquid solution. Our results show substantial incorporation of glycine and alanine into ices VI and VII via structural cogrowth with partition coefficients higher than 0.1. These data imply that in such oceans, the interaction of potential water plumes within the ice mantle would involve partitioning of amino acids into high-pressure ices, possibly complicating their delivery from the ice-silicate boundary upward to the subsurface ocean. The moderate incompatibility of amino acids with high-pressure ices adds another dimension of complexity to the availability of biomolecules for potential life and thus habitability in large extraterrestrial ocean worlds.

## MATERIALS AND METHODS

### High temperature-pressure experiment

Before the experiments, we prepared aqueous solutions of glycine and alanine (1 mol kg^−1^ H_2_O; BioUltra purity; >99%, Aldrich) using Milli-Q water (resistivity 18 MΩ·cm). We used relatively high concentrations of amino acids to achieve good quality and signal-to-noise ratio of the Raman spectra. There are very few observational constraints on inorganic salts in ocean worlds, with the exception of Enceladus’ ocean, which, according to Cassini’s Cosmic Dust Analyzer, is mainly composed of NaCl (with relatively less KCl and NaHCO_3_ or Na_2_CO_3_). Here, we used a rather high concentration of NaCl (=0.55 mol kg^−1^; approximately equal to the salinity of modern Earth’s seawater in which salts are continuously supplied by continental weathering) as the background electrolyte environment. Large ocean worlds may have a more diluted water chemistry than the terrestrial ocean (*94*, *95*), given the much higher bulk water content (>10 wt % H_2_O compared with <0.1 wt % for Earth) as well as no or limited continental weathering supply (*38*). Moreover, as elaborated later in this section, NaCl concentration (between 0 and 1 mol kg^−1^ H_2_O) has little, if any, effect on our results. We did not adjust the pH given the poorly constrained pH values of large extraterrestrial oceans. Thus, glycine and alanine were in their zwitterionic forms and should have remained net neutral under the investigated *T*-*P* conditions (*96*).

Our high *T*-*P* experiments were carried out in an externally heated DAC (*97*) composed of two identical diamond anvils with a 500-μm culet in diameter. The sample chamber (∼200 μm in diameter) was prepared using a preindented T-301 stainless steel gasket (∼70 μm in thickness). The gasket was drilled and lined with gold (and drilled again within the gold filling) to avoid any reaction between the aqueous solution and potentially catalytic steel walls at high *T*-*P*. Three small ruby spheres and subsequently one microdroplet (∼50 nl) of amino acid solution (1 mol kg^−1^ H_2_O) were loaded into the sample chamber laid atop of one diamond anvil and then quickly enclosed by the other. During the experiment, the DAC was heated by an externally coated resistive coil. The real-time temperature of the sample was continuously monitored using a commercially calibrated K-type thermocouple in contact with the gasket and one anvil with an uncertainty ±0.4°C. The target pressure was precisely modulated with a piston acting on the DAC through a deformable membrane pressurized by He gas. The in situ pressure was estimated by analyzing the red fluorescence of Cr^3+^-bearing ruby spheres (*98*). The emission line of a neon (Ne) lamp was collected along with every measurement of ruby as an internal absolute calibration. The shift of the R1 fluorescence line of ruby is quantitatively dependent on *T*-*P* (*99*, *100*). For the present batch of ruby spheres, the temperature dependence of the R1 shift was well calibrated, and the data were best fitted with a second-order polynomial equation (*41*). The uncertainty of the pressure measurement is within 50 MPa (table S2). Neither dissolution nor alteration of the ruby spheres was observed up to the highest *T*-*P* investigated in this study. This experimental setup is identical to that used by Journaux *et al.* (*41*), who successfully measured the melting curves of ices VI and VII.

During each experiment, we initially increased pressure at ambient (room) temperature until the complete crystallization of the solution as ice VI. Then, pressure was gradually decreased to melt most of the ice, leaving a small single crystal (<20 μm) as a seed during the experiments. Temperature and pressure have counteractive effects on the growth of high-pressure polymorphs of water ice: increasing temperature melts ice while increasing pressure grows ice (the *P* dependence is the opposite of that of ice Ih). At a given temperature, we slowly increased the pressure to grow a single ice crystal. Before any measurements, the large crystal was kept for at least 20 min at constant *T*-*P* to allow the partitioning equilibrium between the ice edge and its coexisting fluid. We then measured Raman spectra of high-pressure ice and fluid near the fluid-ice boundary using the same settings (laser power and acquisition parameters, including time and focusing; see below for the detailed parameters). Figure 1 shows one example of a measurement and the corresponding Raman spectra. The distance of two points corresponds to one euhedral face of the tetragonal (pseudo hexagonal) ice VI crystal. The crystal is in contact with the upper anvil near the red point and extends obliquely toward the opposing anvil, such that the liquid contribution at the solid measurement point is minimized. To check the homogeneity of partitioning, we collected Raman spectra of liquid-ice pairs in different locations around the large crystal at a given *T*-*P*, before increasing temperature and then pressure for another round of melting-freezing and analyses. The whole system eventually became overpressurized with respect to ice VII, leading to the bulk crystallization of ice VII. Pressure was then slowly decreased to select a small single crystal of ice VII for a repeat of the previous steps. During the experiment, ice VI and VII crystals were always denser than their coexisting aqueous solution, and we did not observe any precipitation of solid glycine or alanine even when the icy crystals grew very large (figs. S3 and S4).

Raman spectra were collected in the 700 to 1200 cm^−1^ and 2800 to 3800 cm^−1^ spectral regions using a confocal LabRam HR800 spectrometer (Horiba Jobin-Yvon) of 800-mm focal length, with a grating of 1800 gr mm^−1^ and a Mitutoyo 50× long working distance objective (0.42 NA). Incident light was generated from a 532-nm Nd:YAG laser (Torus Laser, Laser Quantum) and attenuated as much as needed to avoid ice melting. Acquisition time was set to be 10 s. Raman spectra were processed with the PeakFit software. A linear background was systematically subtracted from the spectra, and the characteristic Raman peaks of glycine or alanine were fitted using a Voigt function. During the data processing, we carefully selected pairs of Raman spectra of ice and liquid to estimate the partition coefficient, primarily based on the relative intensity and shape of O─H symmetric stretching peaks of the ices, which are very sensitive to any liquid contribution (Fig. 1).

Partition coefficients of amino acids (AA) were estimated as the area ratio of the Raman characteristic peaks of glycine or alanine between ice VI or VII and aqueous solution. The Raman peak area of a dissolved species *i* in phase *a* (*S_i,a_*) is proportional to its concentration in phase *a* (*C_i,a_*), the Raman cross section σ*_i,a_*, and a transfer function *F* (depends on the characteristics of the spectrometer and acquisition conditions of the Raman spectrometer), assuming that there is no disturbance of other species on the Raman contribution of *i,a*

$$S_{i,a}=F\;\sigma_{i,a}\;C_{i,a}$$ (1)

We measured Raman spectra of 1 mol kg^−1^ glycine and alanine solutions over a range of NaCl concentrations between 0.1 and 1 mol kg^−1^, and we observed no detectable effect of the salt concentration on the Raman signature of amino acids. Our observation agrees well with the very limited salting-out effect expected for glycine and alanine in a previous study (*101*), further validating our assumption that $\sigma_{\text{AA},\text{fluid}}$ does not depend on NaCl concentration within the relevant range. Previous studies also noted linear correlations (Beer’s law behavior) between concentration of organics and Raman intensity of their characteristic peaks in aqueous solutions, including amino acids, over a wide range of concentration, using either an internal or external standard [e.g., Aarnoutse and Westerhuis (*102*); Numata *et al.* (*103*); Picard *et al.* (*104*)]. Here, the limits of detection and quantification were conservatively evaluated as the concentration corresponding to a signal thrice (3 σ_back_) and 10 times (10 σ_back_) the SD of the background signal, i.e., 0.03 and 0.1 mol kg^−1^ AA, respectively (*105*).

The exact values of σ_*i*,AA_ in aqueous and high-pressure ices are hard to constrain. For example, both the molecular vibrational environment and possible crystal-orientation effects in the high-pressure ice phase may influence the Raman signal intensity. However, we compared the Raman spectra of glycine and alanine in aqueous and high-pressure ices and observed no differences in wave number and peak shape (fig. S5). Moreover, our first-principles calculations suggest similar coordination properties of amino acids in aqueous and high-pressure ice phases. These results suggest that the local hydration environment of glycine is not drastically different between liquid water and high-pressure ice phases. In addition, both liquid water and high-pressure ices are characterized by dynamic hydrogen disorder, which may be further enhanced by dissolved solutes such as salts. We therefore infer that the vibrational environment of amino acids in liquid water, ice VI, and ice VII is broadly comparable, although not necessarily identical. During the experiments, we measured Raman spectra at different edges/positions of the high-pressure crystals together with the adjacent aqueous phases. The resulting spectra were similar and did not show systematic position-dependent differences (figs. S3 and S4). These observations suggest that crystal orientation effects are minor, if present, under our measurement conditions. This implies that the incorporation of glycine or alanine into ice VI and VII does not substantially change either the conformation or the charge of the molecule between these phases. Therefore, it is reasonable to assume that the Raman cross section σ_AA_ is not drastically different in the three phases of interest here, i.e., the aqueous solution, ice VI, and ice VII, within the *T*-*P* range of interest.

Thus, the partition coefficient $K_{p}$ of an amino acid between ice VI/VII and fluid can be approximated as follows

$$K_{p,\text{AA}}^{\text{ice}-l}=\frac{C_{\text{AA},\text{ice}}}{C_{\text{AA},\text{liquid}}}=\frac{S_{\text{AA},\text{ice}}}{S_{\text{AA},\text{liquid}}}$$ (2)

Amino acids (including glycine and alanine) usually have several characteristic peaks in their Raman spectra. To obtain the highest possible accuracy of *K*_*p*,AA_, we selected the characteristic Raman peaks with high intensity, lack of interference with other bands, and a linear background. Here, we chose the homologous Raman peaks of glycine and alanine, at 897 and 848 cm^−1^, respectively. The characteristic peak of glycine at 897 cm^−1^ at ambient *T*-*P* is assigned to the scissoring signal of the carboxylate group δ(COO^−^) and the stretching of the glycine backbone ν(C─C). The characteristic peak of alanine at 848 cm^−1^ has an additional contribution from the stretching of an additional C─N mode in the molecule ν(C─N) (*40*, *103*, *106*, *107*).

### First-principles calculations

First-principles calculations were performed to investigate the evolution of the hydrogen bond network between amino acids and water molecules during the crystallization of ices VI and VII. All simulations were conducted using the Vienna Ab initio Simulation Package (VASP 5.4.4) (*108*). The exchange-correlation interactions were treated using the Perdew-Burke-Ernzerhof functional within the generalized gradient approximation (*109*). The projector augmented wave method (*110*) was used to describe core electrons, and van der Waals interactions were included via the DFT-D3(BJ) dispersion correction scheme (*111*). A plane-wave cutoff energy of 600 eV was used. Brillouin zone sampling was restricted to the Γ-point due to the large supercell sizes (3 × 2 × 2 for ice VI and 4 × 4 × 4 for ice VII).

To explore the dynamics of amino acid–water interactions, MD simulations were performed under the NVT (*112*) ensemble (constant number of particles, volume, and temperature). We adopted 116 water molecules and 1 amino molecule for ice VI simulations (corresponding to 0.86 mol % or 0.48 mol kg^−1^ H_2_O) and 124 water molecules and 1 amino molecule for ice VII simulations (corresponding to 0.81 mol % or 0.45 mol kg^−1^ H_2_O). Two reverse annealing MD simulations were initially conducted: one for ice VI, heating from −48° to 152°C at a rate of 0.02°C per step, and another for ice VII, heating from −50° to 200°C at 0.02°C per step. The crystallographic space groups were set to *P*4_2_/*nmc* for ice VI and *Pn*$\bar{3}$*m* for ice VII, with lattice parameters derived from experimental data (*113*, *114*): *a* = *b* = 6.181 Å, *c* = 5.698 Å for ice VI, and *a* = *b* = *c* = 3.3 Å for ice VII. The hydrogen orderings of ices VI and VII are based on the first-principles study of Kuo and Kuhs (*115*) and Kuo and Klein (*116*). Four water molecules (H_2_O) were replaced with one alanine (C_3_H_7_NO_2_) or one glycine (C_2_H_5_NO_2_) molecule, given the comparable number of atoms.

To equilibrate the substituted structures, four NVT MD simulations were conducted: two at 152°C for ice VI with alanine or glycine, and two at 200°C for ice VII with alanine or glycine. Each simulation ran for 3000 steps with a time step of 0.25 fs. System pressure, temperature, and energy were monitored every 1000 steps to ensure thermodynamic equilibrium. Subsequently, 12 annealing MD simulations were conducted with varying initial configurations. For ice VI with alanine or glycine, six simulations (three per amino acid) were performed, cooling from 152° to 52°C at 0.01°C per step. For ice VII with alanine or glycine, six simulations (three per amino acid) were also conducted, cooling from 200° to 100°C at 0.01°C per step. Following annealing, 12 NVT-ensemble simulations were carried out to equilibrate the annealed structures. The average pressures and temperatures were 3.8 GPa and 52°C for alanine in ice VI, 3.4 GPa and 52°C for glycine in ice VI, 5.1 GPa and 100°C for alanine in ice VII, and 4.6 GPa and 100°C for glycine in ice VII, respectively. In all MD simulations, half of the water molecules were fixed in their ice VI or ice VII lattice positions, while the remaining water molecules and amino acids were allowed to move freely.

Structural optimizations were performed on the equilibrated systems to analyze the hydrogen bonding network between amino acids and the surrounding ice. To quantify the hydrogen bonding interactions, partial radial distribution functions (PRDFs) (*117*) were calculated from the 12 NVT simulations at both high (152°C for ice VI; 200°C for ice VII) and low (52°C for ice VI; 100°C for ice VII) temperatures. Specifically, PRDFs were calculated between nitrogen and oxygen atoms of the amino acids and hydrogen atoms from water, as well as between hydrogen atoms of the amino acids and oxygen atoms from water. The oxygen atoms were labeled as O1 (carbonyl group) and O2 (hydroxyl group), while the hydrogen atoms were labeled as H1 and H2 (amino group) and H3 (hydroxyl group). The PRDF was defined as

$$\text{PRDF}\left(R\right)=\frac{N}{\rho_{0}N_{a}N_{b}}{\left\langle \sum_{i,j}\delta \left(\vec{r}-|\vec{r_{i}}-\vec{r_{j}}|\right)\right\rangle }_{t}$$ (3)

where *N* is the total number of atoms; ρ_0_ is the atomic number density; *N_a_* and *N_b_* are the numbers of atoms of types *a* and *b*, respectively; *i* ranges from 1 to *N_a_*; *j* ranges from 1 to *N_b_*; $\vec{r}$ represents atomic coordinates; and ⟨…⟩*_t_* denotes time averaging over the simulation.

The coordination number (*CN*) was computed by integrating the PRDF

$$\text{CN}\left(R\right)=\int_{0}^{R}4\pi r^{2}\rho_{0}\text{PRDF}\left(R\right)\mathrm{dr}$$ (4)

For analysis of amino acid–ice interactions, only the coordination numbers for N─, O (from amino acids)─H, and H (from amino group)─O (from water) pairs were considered.

## Supplementary Materials

### This PDF file includes:

Supplementary Text Figs. S1 to S8 Tables S1 and S2 References
