# Supplementary material for: How high-pressure ices on large ocean worlds can support habitability: The compatibility of amino acids into ice VI and VII
Source: Sci Adv. 2026 Oct 21;12(43):eaec2045. doi: 10.1126/sciadv.aec2045 (PMC13685913; doi:10.1126/sciadv.aec2045)
Supplement: Supplementary file 1 [file sciadv.aec2045_sm.pdf]

Science Advances
AAAS

Supplementary Materials for

How high-pressure ices on large ocean worlds can support habitability: The compatibility of amino acids into ice VI and VII

Jihua Hao *et al.*

Corresponding author: Jihua Hao, hao@ustc.edu.cn; Can Liu, can2021@mail.ustc.edu.cn; Feixiang Liu, liufeixiang@smail.nju.edu.cn; Isabelle Daniel, isabelle.daniel@univ-lyon1.fr

Sci. Adv. **12**, eaec2045 (2026)
DOI: 10.1126/sciadv.aec2045

This PDF file includes:

Supplementary Text
Figs. S1 to S8
Tables S1 and S2
References

Supplementary Text 1: Phase Diagram of Water

Here, we report the temperature and pressure conditions of our measurements in the phase diagram of water (Fig. S1, the melting curves is from Journaux et al. (*23*)). In addition, we also display the pressure-temperature of the liquidus obtained during our measurements (Fig. S1). The systematic departure from the liquidus of pure water or salty water has been confirmed by several experiments, likely reflecting non-ideal solute partitioning, strong hydration of amino acids, and pressure-dependent changes in the relative stability of liquid water and high-pressure ice phases. However, the number of data points doesn't yet allow to define a curve and propose a thermodynamic model of the effect of glycine or alanine on the meting curve of ice VI and VII. This is beyond the scope of the present contribution.

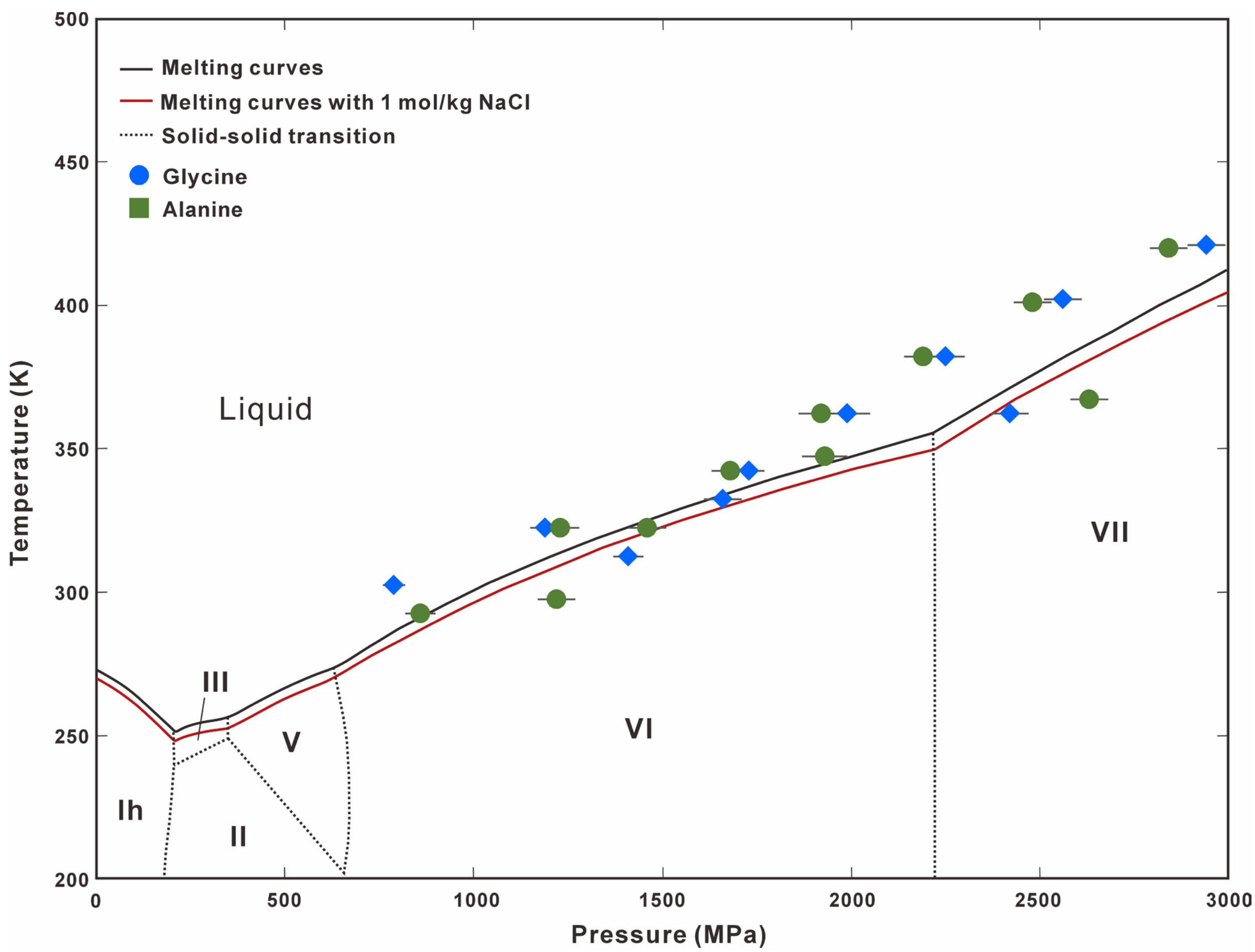

Fig. S1. Phase diagram of water as the function of temperature and pressure. The blue diamonds and green circles indicate the pressure-temperature conditions of our experiments.

Supplementary Text 2: Solubility of Glycine and Alanine at Elevated Temperatures and Pressures

In this note, we compile and plot the reported solubilities of glycine and alanine at temperatures and pressures higher than ambient values (**Fig. S2**). Solubility of glycine and alanine increases monotonically with temperature and under the water-saturation pressure, solubility of glycine and alanine can reach approximately 19 and 7 mole kg^{-1} H_2O respectively (**Fig. S2A**). Compared with temperature, pressure has no or slightly negative effects on solubilities of glycine and alanine (**Fig. S2B**).

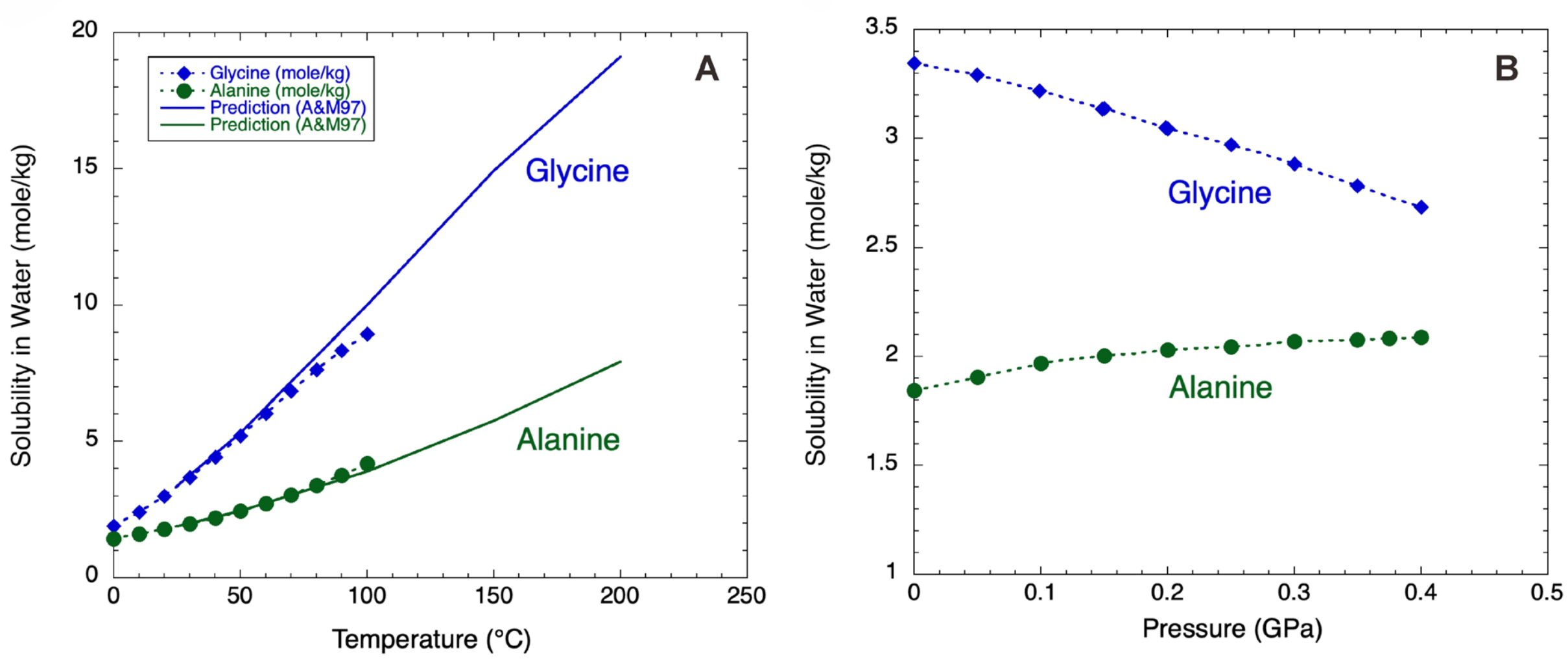

Fig. S2. Solubility of glycine (blue) and alanine (green) at elevated (A) temperatures (and corresponding water-saturation pressure) and (B) pressures (and 25 °C). Data sources: experimental results (blue diamond and green circle connected with dashed lines) from Lundblad & Macdonald (*118*) and Matsuo et al. (*119*), theoretical prediction (solid lines) using thermodynamic properties of amino acids from Amend & Shock (*15*).

Supplementary Text 3: Representative Raman Spectra of Amino Acid Solutions Equilibrated with High-pressure Ices

In this note, we provide several pairs of representative Raman spectra collected in our experiment. The major criterion is to have high intensity of the OH-stretching peak at 3200 cm^{-1} (for ice VI) or 3300 cm^{-1} (for ice VII) relative to that of the OH-stretching peak at 3400 cm^{-1} (for liquid water), indicating no/little contribution of liquid water to the signal of ices.

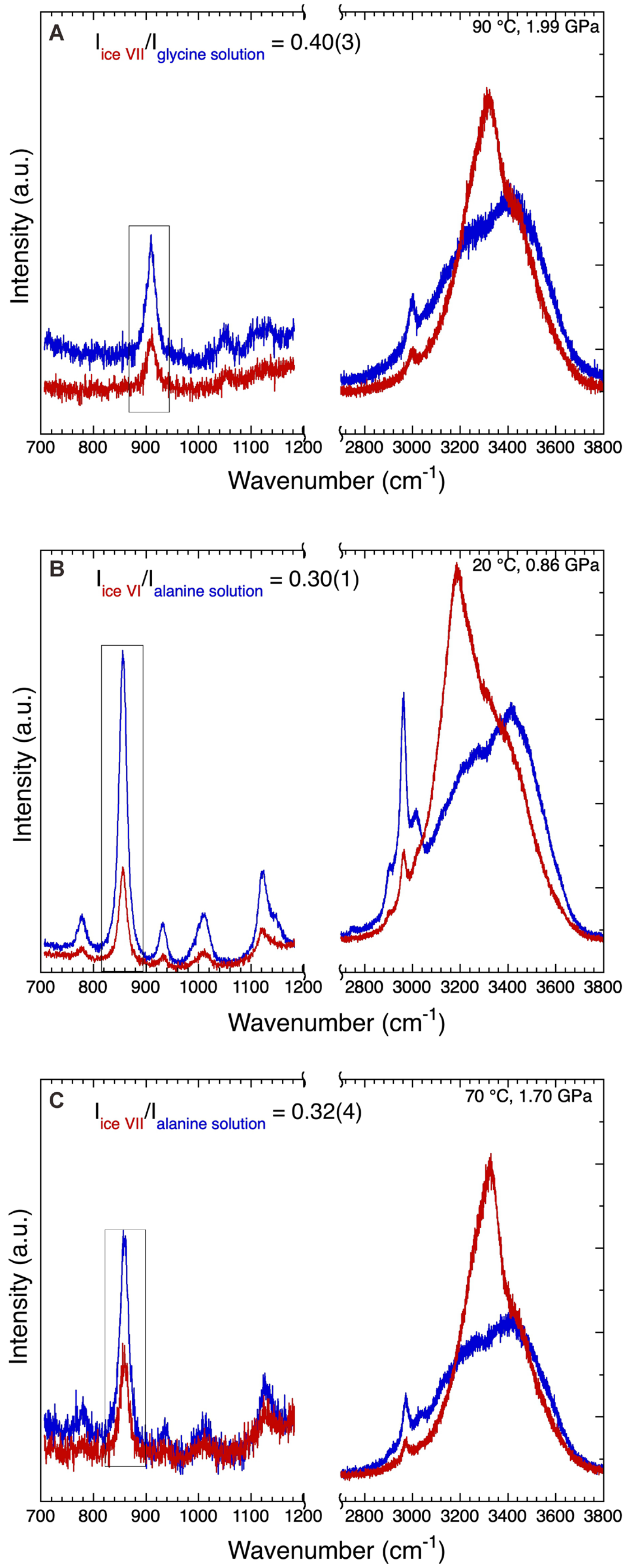

A
I ice VII/I glycine solution = 0.40(3)
90 °C, 1.99 GPa
Intensity (a.u.)
700 800 900 1000 1100 1200
2800 3000 3200 3400 3600 3800
Wavenumber (cm-1)
B
I ice VI/I alanine solution = 0.30(1)
20 °C, 0.86 GPa
Intensity (a.u.)
700 800 900 1000 1100 1200
2800 3000 3200 3400 3600 3800
Wavenumber (cm-1)
C
I ice VII/I alanine solution = 0.32(4)
70 °C, 1.70 GPa
Intensity (a.u.)
700 800 900 1000 1100 1200
2800 3000 3200 3400 3600 3800
Wavenumber (cm-1)

Fig. S3. Raman spectra of 1 mol kg^{-1} solutions of amino acids (blue) equilibrated with high-pressure ices (red). (A) ice VII and its co-existing glycine solution at 90 °C and 1.99 GPa; (B) ice VI and its co-exiting alanine solution at 20 °C and 0.86 GP; (C) ice VII and its co-exiting alanine solution (blue) at 70 °C and 1.70 GPa. The rectangle boxes mark the band, whose relative intensity was used to calculate the partition coefficient. It is assigned to the scissoring of the carboxylic group δ(COO-) and the stretching of glycine and alanine carbon backbone ν(C–C); the alanine peak has an additional contribution from the stretching of the additional C–N in the molecule ν(C–N).

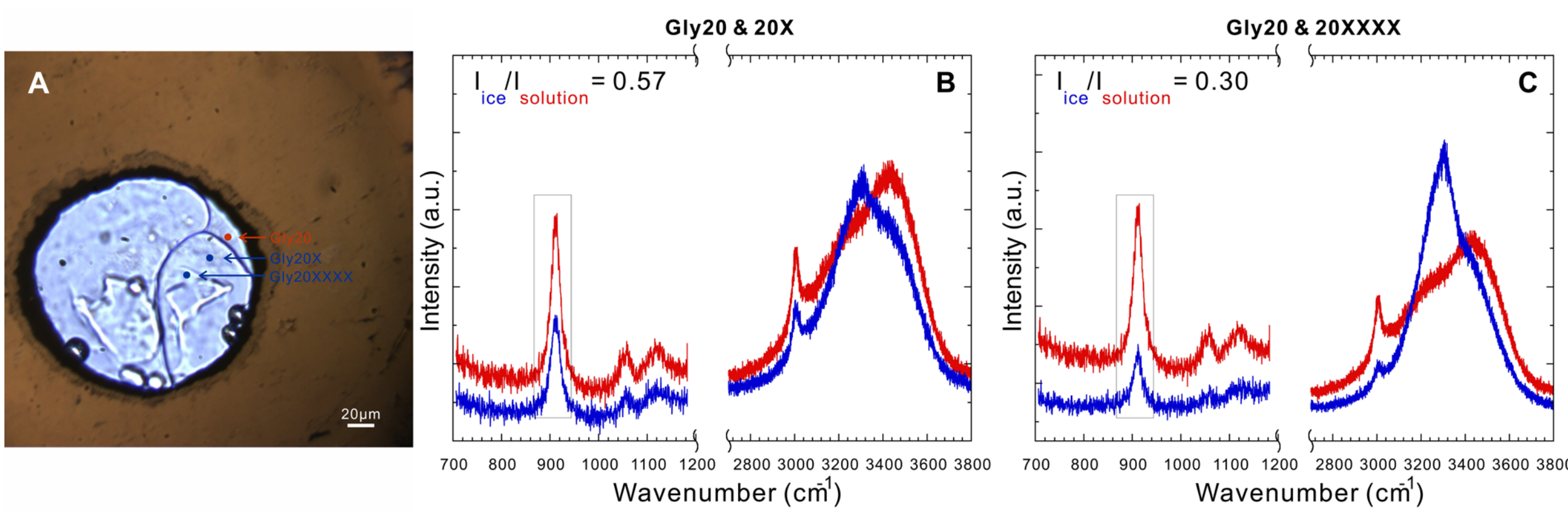

Fig. S4. Raman spectra of 1 mol kg^{-1} glycine solution (red) and ice VII (blue) at large extent of freezing. Here we show the Raman signal in different locations of ice VII at 149 °C, 2.94GPa. (A) Image of the sample in the diamond anvil cell; transmitted light, scale bar: 20 μm. Red and blue dots show the locations where Raman spectra were typically measured in the fluid and ice VII phase, respectively. (B) and (C) show the Raman spectra corresponding to different locations. The box centered at 900 cm^{-1} shows the characteristic peak of glycine.

Supplementary Text 4: Partition Coefficients of Glycine and Alanine into Ice VI and VII.

In this note, we summarize the measurement on the partition coefficients of glycine and alanine into ice VI and VII at various temperatures and pressures. Here, we select the most prominent characteristic peak of amino acid to estimate the partition coefficient. Under the T-P conditions of this study, glycine has three apparent characteristic peaks, i.e., C-C stretching vibration band at 900 cm^{-1}, C-N stretching vibration band at 1040 cm^{-1}, and C-H symmetric stretching band at 2980 cm^{-1} (*57*, *120*). Among the three peaks, C-C stretching band has the strongest intensity and the only apparent one in ice sample (**Fig. 1B, S3A**). Therefore, area of C-C stretching band is used to calculate the partition coefficient.

Alanine has more characteristic peaks than glycine, for example, H-N-C and C-C-H bending band at 780 cm^{-1}, C-N and C-C stretching band at 850 cm^{-1}, and C-N-H bending band at 925 cm^{-1} (*121*). However, the 850 cm^{-1} peak is the strongest and most differentiable one (**Fig. S3B, C**), allowing to estimate the partition coefficient with lowest uncertainty. Therefore, we used the peak area of C-N and C-C stretching band (850 cm^{-1}) to calculate the partition coefficient of alanine.

Table S1. Raw data of selected Raman measurements with minimal or no contribution of fluid composition based on the O-H stretching bond of water. All partition coefficient values were estimated by the ratio of amino acid peak areas between ice and water phases.

Phase	Sample	Temperature (°C)	Pressure (GPa)	Amplitude	Peak Area	Kp_{AA}^{ice-l}	FWHM	Std Error
Glycine								
Liquid	Gly02	30	0.79 ±0.03	1236.26	34860.18		19.40	0.23
Ice VI	Gly02xx	30	0.79 ±0.03	204.16	5610.32	0.16	19.90	0.80
Liquid	Gly03	50	1.19 ±0.04	1090.24	31850.87		20.02	0.28
Ice VI	Gly03xxx	50	1.19 ±0.04	264.82	7644.82	0.24	19.52	0.90
Liquid	Gly04	50	1.19 ±0.04	1095.19	31829.70		20.02	0.27
Ice VI	Gly04x	50	1.19 ±0.04	216.26	6008.59	0.19	19.91	0.93
Liquid	Gly05	70	1.73 ±0.04	1007.23	31342.77		20.92	0.35
Ice VI	Gly05xx	70	1.73 ±0.04	67.94	2135.66	0.07	21.46	3.22
Liquid	Gly06	70	1.73 ±0.04	1012.35	31470.48		21.67	0.31
Ice VI	Gly06xx	70	1.73 ±0.04	103.45	3222.92	0.10	20.17	3.64
Ice VI	Gly06xxx	70	1.73 ±0.04	66.32	1877.37	0.06	21.72	2.39
Liquid	Gly07	90	2.42 ±0.05	893.83	30891.24		23.30	0.43
Ice VI	Gly07xx	90	2.42 ±0.05	172.52	5608.36	0.18	23.22	1.35
Liquid	Gly08	90	2.42 ±0.05	960.38	33056.60		23.00	0.43
Ice VI	Gly08x	90	2.42 ±0.05	102.04	2899.10	0.09	22.93	1.66
Ice VI	Gly08xx	90	2.42 ±0.05	81.18	2269.76	0.07	21.06	2.11

Liquid	Gly09	90	2.42 ±0.05	987.84	33642.44		22.67	0.51
Ice VI	Gly09xx	90	2.42 ±0.05	144.21	4733.54	0.14	21.54	2.77
Liquid	Gly11	90	1.99 ±0.06	408.40	11923.35		20.47	0.74
Ice VII	Gly11x	90	1.99 ±0.06	176.59	4699.16	0.39	18.47	1.49
Ice VII	Gly11xx	90	1.99 ±0.06	170.16	4725.64	0.40	20.18	1.39
Liquid	Gly12	90	1.99 ±0.06	395.99	12001.33		22.25	0.65
Ice VII	Gly12x	90	1.99 ±0.06	161.40	5029.69	0.42	19.85	16.40
Liquid	Gly16	130	2.56 ±0.05	619.84	19942.39		22.58	0.56
Ice VII	Gly16x	130	2.56 ±0.05	380.78	12480.75	0.63	22.92	1.02
Ice VII	Gly16xx	130	2.56 ±0.05	367.85	12134.83	0.61	22.62	0.96
Liquid	Gly17	130	2.56 ±0.05	628.74	20846.79		22.65	0.68
Ice VII	Gly17x	130	2.56 ±0.05	391.94	12181.08	0.58	22.66	0.77
Liquid	Gly20	149	2.94 ±0.05	631.35	21352.94		22.57	0.78
Ice VII	Gly20x	149	2.94 ±0.05	356.31	12209.45	0.57	24.11	0.92
Ice VII	Gly20xxxx	149	2.94 ±0.05	205.78	6463.38	0.30	21.11	1.76
Liquid	Gly21	169	3.27 ±0.04	527.42	19301.67		24.74	1.00
Ice VII	Gly21xxx	169	3.27 ±0.04	241.99	8064.27	0.42	23.49	1.42
Liquid	Gly22	60	1.66 ±0.05	1449.35	47907.59		22.13	0.29
Ice VII	Gly22x	60	1.66 ±0.05	444.90	13782.08	0.29	21.60	0.62
Ice VII	Gly22xx	60	1.66 ±0.05	290.86	9102.80	0.19	21.89	0.94
Ice VII	Gly22xxx	60	1.66 ±0.05	275.95	9464.76	0.20	22.14	2.42
Liquid	Gly23	60	1.66 ±0.05	1131.43	35003.57		21.75	0.28
Ice VII	Gly23x	60	1.66 ±0.05	385.91	12456.75	0.36	20.81	1.63
Liquid	Gly24	60	1.66 ±0.05	1362.52	43479.28		21.74	0.27
Ice VII	Gly24xx	60	1.66 ±0.05	595.35	18452.43	0.42	21.68	0.42
Liquid	Gly25	40	1.41 ±0.04	966.30	29680.60		20.70	0.44
Ice VII	Gly25x	40	1.41 ±0.04	211.31	6709.55	0.23	22.15	1.09
Ice VII	Gly25xxx	40	1.41 ±0.04	179.00	5664.21	0.19	22.63	1.36
Liquid	Gly26	40	1.41 ±0.04	858.22	26077.96		21.31	0.33
Ice VII	Gly26x	40	1.41 ±0.04	227.31	6974.22	0.27	21.73	1.17
Alanine								
Liquid	Ala02	20	0.86 ±0.04	2533.93	64160		17.67	0.15

Ice VI	Ala02X	20	0.86 ±0.04	766.76	19451	0.30	17.43	0.43
Ice VI	Ala02XXX	20	0.86 ±0.04	645.64	16466	0.26	17.24	0.48
Liquid	Ala03	50	1.23 ±0.05	1751.69	46441		18.08	0.23
Ice VI	Ala03x	50	1.23 ±0.05	343.45	8932	0.19	17.93	0.79
Ice VI	Ala03XX	50	1.23 ±0.05	146.87	3838	0.08	19.03	1.29
Ice VI	Ala03XXX	50	1.23 ±0.05	248.60	6263	0.13	18.61	0.85
Liquid	Ala04	75	1.93 ±0.06	1548.23	43518		19.51	0.24
Ice VI	Ala04XX	75	1.93 ±0.06	266.22	7196	0.17	18.96	0.96
Liquid	Ala05	75	1.93 ±0.06	1546.92	43035		19.44	0.26
Ice VI	Ala05X	75	1.93 ±0.06	437.45	11913	0.28	19.14	0.66
Liquid	Ala06	95	2.63 ±0.05	1468.34	43053		20.61	0.30
Ice VI	Ala06XX	95	2.63 ±0.05	292.55	8078	0.19	20.21	0.87
Liquid	Ala12	129	2.48 ±0.05	600.56	17230		19.76	0.74
Ice VII	Ala12X	129	2.48 ±0.05	387.58	11155	0.65	19.56	1.05
Ice VII	Ala12XX	129	2.48 ±0.05	352.85	10027	0.58	20.79	1.06
Liquid	Ala13	129	2.48 ±0.05	615.80	17866		20.01	0.70
Ice VII	Ala13X	129	2.48 ±0.05	369.46	10616	0.59	19.82	1.01
Liquid	Ala14	148	2.84 ±0.05	622.40	18341		20.98	0.67
Ice VII	Ala14X	148	2.84 ±0.05	214.74	5682	0.31	19.91	1.14
Liquid	Ala15	148	2.84 ±0.05	645.42	19044		20.00	0.72
Ice VII	Ala15X	148	2.84 ±0.05	269.27	8450	0.44	20.88	1.87
Ice VII	Ala15XX	148	2.84 ±0.05	204.14	5161	0.27	19.85	1.07
Liquid	Ala17	172	3.37 ±0.06	757.34	23643		21.21	0.68
Ice VII	Ala17XX	172	3.37 ±0.06	263.11	7772	0.33	21.45	1.10
Ice VII	Ala17XXX	172	3.37 ±0.06	436.17	12437	0.53	21.68	0.69
Liquid	Ala19	70	1.68±0.05	914.47	24876		18.50	0.55
Ice VII	Ala19X	70	1.68±0.05	361.60	9657	0.39	18.20	1.04
Ice VII	Ala19XXX	70	1.68±0.05	335.45	8310	0.33	18.42	0.84
Liquid	Ala20	70	1.68±0.05	486.03	13159		19.85	0.60
Ice VII	Ala20X	70	1.68±0.05	193.54	4160	0.32	18.27	1.03
Liquid	Ala22	90	1.92±0.06	483.18	12748		18.31	0.63
Ice VII	Ala22XX	90	1.92±0.06	93.82	2085	0.16	19.40	1.66
Liquid	Ala26	25	1.22 ±0.05	1103.93	29188		18.30	0.29
Ice VII	Ala26X	25	1.22 ±0.05	310.33	7802	0.27	18.69	0.68

Ice VII	Ala26XXX	25	1.22 ±0.05	293.06	7726	0.26	18.72	0.71
Liquid	Ala27	25	1.22 ±0.05	1105.77	29445		18.55	0.26
Ice VII	Ala27X	25	1.22 ±0.05	196.50	4677	0.16	18.32	0.79
Ice VII	Ala27XX	25	1.22 ±0.05	226.47	5596	0.19	18.20	0.84
Ice VII	Ala27XXX	25	1.22 ±0.05	261.84	5635	0.19	17.38	0.60
Liquid	Ala28	50	1.46 ±0.05	1141.35	30943		18.70	0.28
Ice VII	Ala28X	50	1.46 ±0.05	320.31	8623	0.28	19.18	0.75
Ice VII	ALa28XX	50	1.46 ±0.05	291.48	7140	0.23	18.41	0.59
Liquid	Ala29	50	1.46 ±0.05	1133.18	30966		19.20	0.29
Ice VII	Ala29XX	50	1.46 ±0.05	373.00	9684	0.31	18.85	0.60
Ice VII	Ala29XXX	50	1.46 ±0.05	463.03	12327	0.40	18.59	0.58

Table S2. Measured partition coefficients of glycine and alanine into ice VI and VII. The relative ice volume was estimated by the relative coverage of ice crystals.

	Temperature (°C)	Pressure (GPa)	Kp_{AA}^{ice-l}	Ice volume
**Glycine**				
Ice VI	30	0.79 ±0.03	0.16	~57%
	50	1.19 ±0.04	0.21 ±0.04	~56%
	70	1.73 ±0.04	0.08 ±0.02	~44%
	90	2.42 ±0.05	0.12 ±0.05	~44%
Ice VII	90	1.99 ±0.06	0.40 ±0.03	~71%
	110	2.25 ±0.05	-	
	130	2.56 ±0.05	0.61 ±0.02	~84%
	149	2.94 ±0.05	0.44 ±0.19	~90%
	169	3.27 ±0.04	0.42	~90%
	60	1.66 ±0.05	0.29 ±0.10	~90%
	40	1.41 ±0.04	0.23 ±0.04	~90%
**Alanine**				
Ice VI	20	0.86 ±0.04	0.28 ±0.03	~93%
	50	1.23 ±0.05	0.14 ±0.05	~63%
	75	1.93 ±0.06	0.22 ±0.08	~63%
	95	2.63 ±0.05	0.19 ±0.04	~63%
Ice VII	129	2.48 ±0.05	0.61 ±0.03	~54%
	148	2.84 ±0.05	0.34 ±0.09	~74%
	172	3.37 ±0.06	0.43 ±0.14	~89%
	70	1.68±0.05	0.35 ±0.04	~56%
	90	1.92±0.06	0.16	~55%
	110	2.19 ±0.05	-	~53%

	25	1.22 ±0.05	0.21 ±0.05	~55%
	50	1.46 ±0.05	0.31 ±0.07	~55%

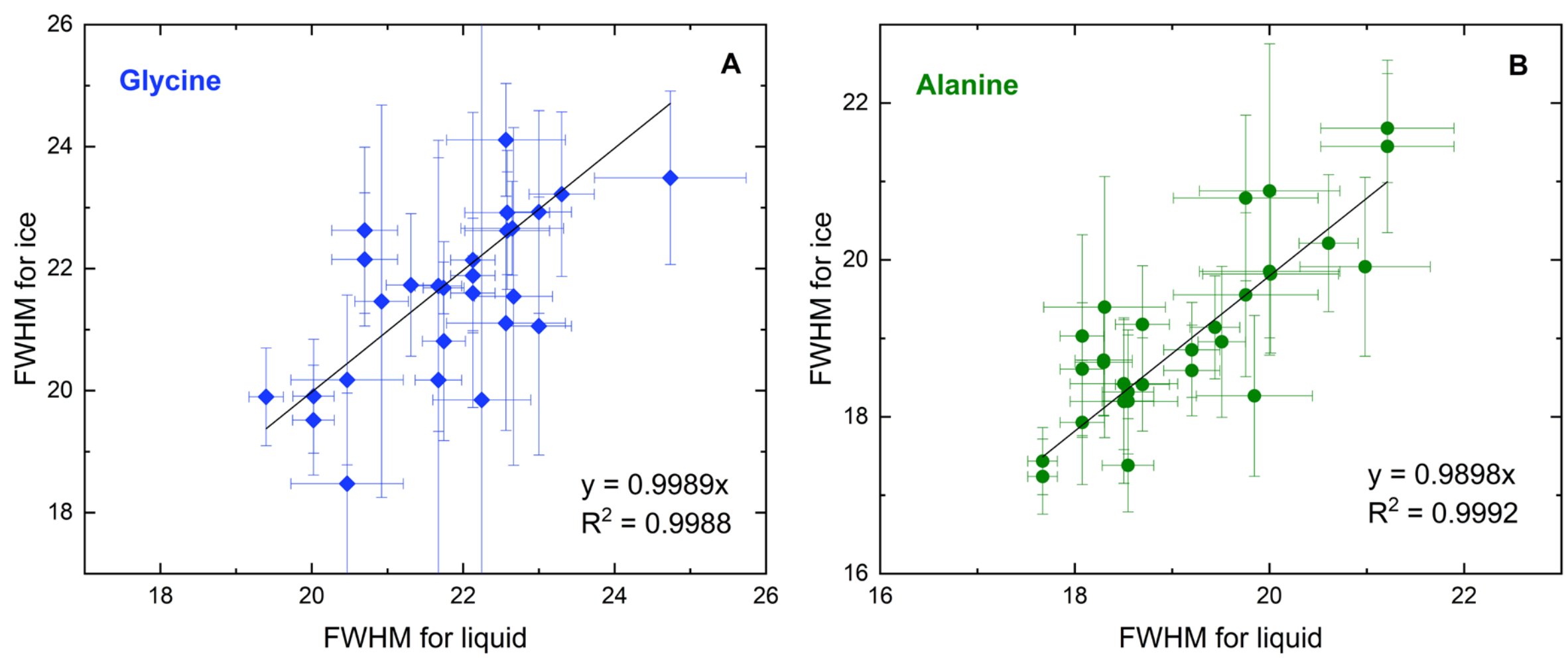

Fig. S5. Correlation of the full width at half-maximum (FWHM) of Raman peaks of (A) glycine and (B) alanine between aqueous and high-pressure ice phases.

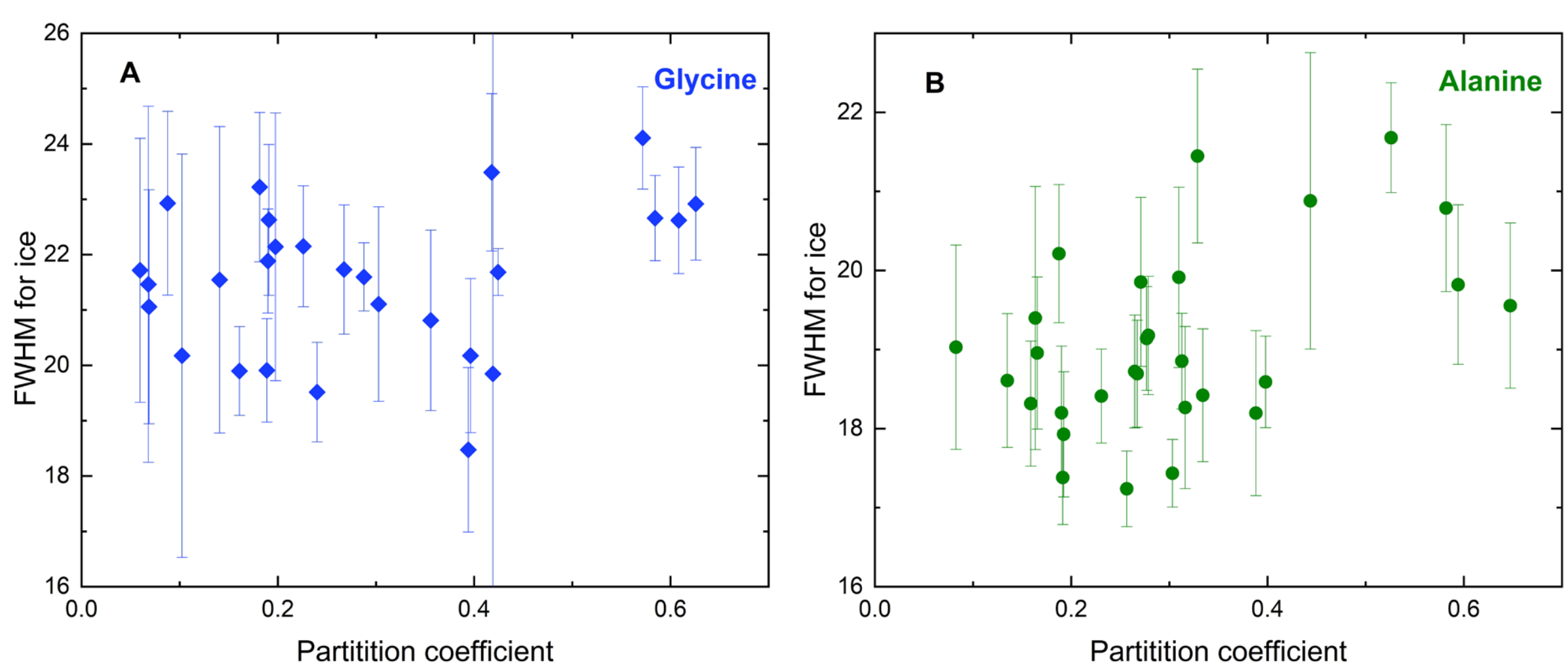

Fig. S6. Plot of the full width at half-maximum (FWHM) of Raman peaks of (A) glycine and (B) alanine in high-pressure ices against the estimated partition coefficients.

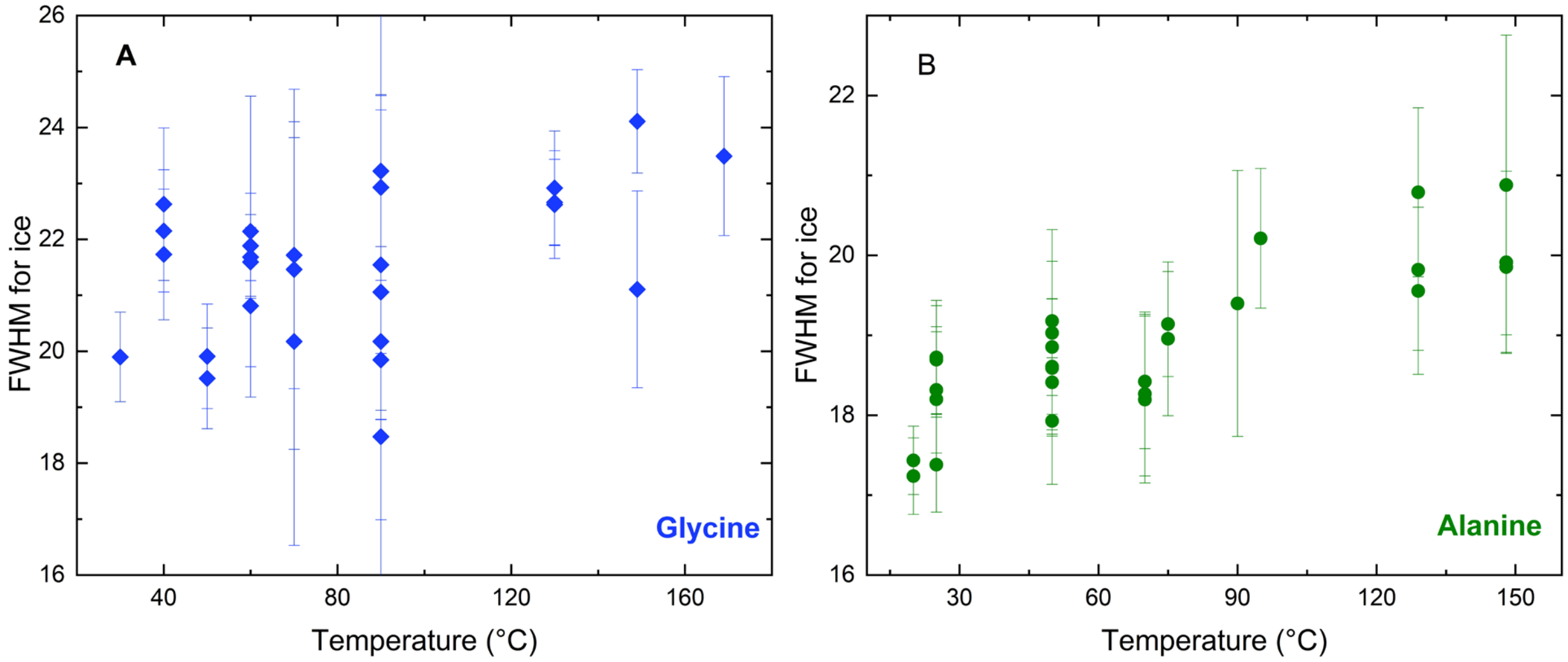

Fig. S7. Plot of the full width at half-maximum (FWHM) of Raman peaks of (A) glycine and (B) alanine in high-pressure ices against temperature.

Supplementary Text 4:

In this text, we show representative structures from molecular dynamics (MD) simulations and static structural optimizations. The MD snapshots are from NVT simulations conducted at various temperatures, and the optimized structures were obtained through energy minimization. Initial models were built based on experimental crystallographic data.

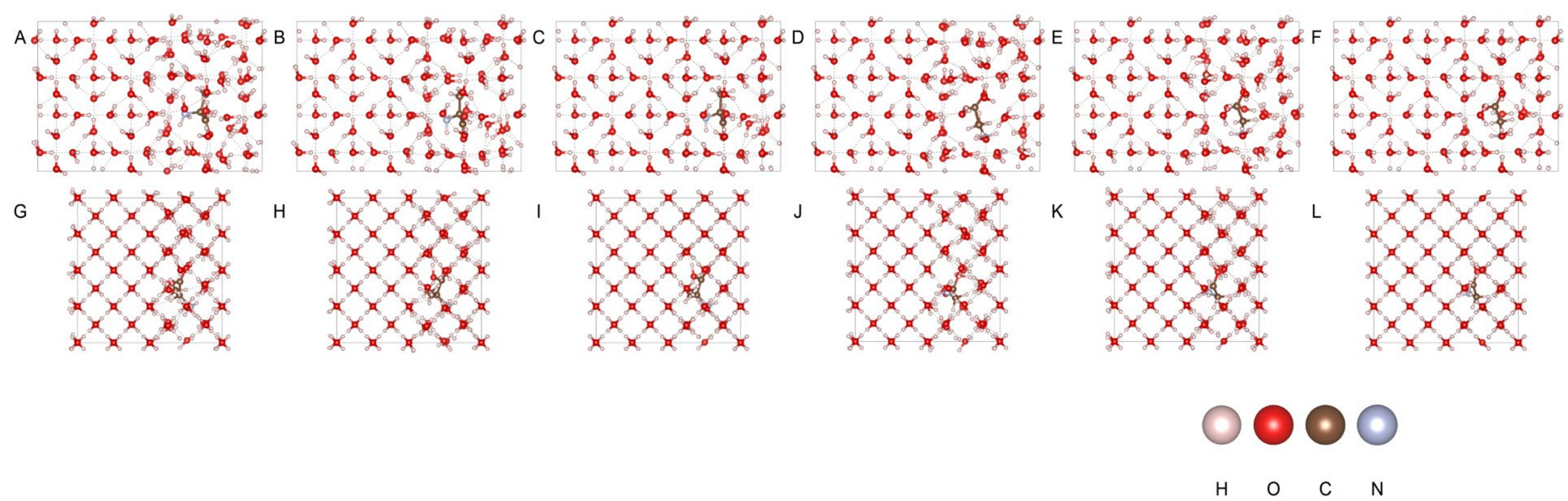

Fig. S8: Representative structures from molecular dynamics (MD) simulations and static structural optimizations. (A–C) Alanine in ice VI: NVT simulations at 152 °C (A) and 52 °C (B), optimized structure (C). (D–F) Glycine in ice VI: NVT simulations at 152 °C (D) and 52 °C (E), optimized structure (F). (G–I) Alanine in ice VII: NVT simulations at 200°C (G) and 100°C (H), optimized structure (I). (J–L) Glycine in ice VII: NVT simulations at 200°C (J) and 100°C (K), optimized structure (L). Atom colors: light pink = H, red = O, brown = C, blue = N.

REFERENCES

1. National Academies of Sciences, Engineering, and Medicine, *Origins, Worlds, and Life: A Decadal Strategy for Planetary Science and Astrobiology 2023–2032* (The National Academies Press, 2023).

2. F. Nimmo, R. T. Pappalardo, Ocean worlds in the outer solar system. *J. Geophys. Res. Planets* **121**, 1378–1399 (2016).

3. NASA Exoplanet Archive, *NASA Exoplanet Science Institute / Caltech IPAC* (2024); https://exoplanetarchive.ipac.caltech.edu/.

4. M. L. Hill, K. Bott, P. A. Dalba, T. Fetherolf, S. R. Kane, R. Kopparapu, Z. Li, C. Ostberg, A catalog of habitable zone exoplanets. *Astron. J.* **165**, 34 (2023).

5. A. Léger, F. Selsis, C. Sotin, T. Guillot, D. Despois, H. Lammer, M. Ollivier, F. Brachet, "A new family of planets? "Ocean Planets"," in *Proceedings of the Conference Towards Other Earths: DARWIN/TPF and the Search for Extrasolar Terrestrial Planets* (ESA Publications Division, 2003), pp. 253–259.

6. R. Heller, R. Pudritz, Water ice lines and the formation of giant moons around super-jovian planets. *Astrophys. J.* **806**, 181 (2015).

7. T. Van Hoolst, G. Tobie, C. Vallat, N. Altobelli, L. Bruzzone, H. Cao, D. Dirkx, A. Genova, H. Hussmann, L. Iess, J. Kimura, K. Khurana, A. Lucchetti, G. Mitri, W. Moore, J. Saur, A. Stark, A. Vorburger, M. Wieczorek, A. Aboudan, J. Bergman, F. Bovolo, D. Breuer, P. Cappuccio, L. Carrer, B. Cecconi, G. Choblet, F. De Marchi, M. Fayolle, A. Fienga, Y. Futaana, E. Hauber, W. Kofman, A. Kumamoto, V. Lainey, P. Molyneux, O. Mousis, J. Plaut, W. Puccio, K. Retherford, L. Roth, B. Seignovert, G. Steinbrügge, S. Thakur, P. Tortora, F. Tosi, M. Zannoni, S. Barabash, M. Dougherty, R. Gladstone, L. I. Gurvits, P. Hartogh, P. Palumbo, F. Poulet, J.-E. Wahlund, O. Grasset, O. Witasse, Geophysical characterization of the interiors of Ganymede, Callisto and Europa by ESA's JUpiter ICy moons Explorer. *Space Sci. Rev.* **220**, 54 (2024).

8. R. T. Pappalardo, B. J. Buratti, H. Korth, D. A. Senske, D. L. Blaney, D. D. Blankenship, J. L. Burch, P. R. Christensen, S. Kempf, M. G. Kivelson, E. Mazarico, K. D. Retherford, E. P. Turtle, J. H. Westlake, B. G. Paczkowski, T. L. Ray, J. Kampmeier, K. L. Craft, S. M. Howell, R. L. Klima, E. J. Leonard, A. Matiella Novak, C. B. Phillips, I. J. Daubar, J. Blacksberg, S. M. Brooks, M. N. Choukroun, C. J. Cochrane, S. Diniega, C. M. Elder, C. M. Ernst, M. S. Gudipati, A. Luspay-Kuti, S. Piqueux, A. M. Rymer, J. H. Roberts, G. Steinbrügge, M. L. Cable, J. E. C. Scully, J. C. Castillo-Rogez, H. C. F. C. Hay, D. M. Persaud, C. R. Glein, W. B. McKinnon, J. M. Moore, C. A. Raymond, D. M. Schroeder, S. D. Vance, D. Y. Wyrick, M. Y. Zolotov, K. P. Hand, F. Nimmo, M. A. McGrath, J. R. Spencer, J. I. Lunine, C. S. Paty, J. M. Soderblom, G. C. Collins, B. E. Schmidt, J. A. Rathbun, E. L. Shock, T. C. Becker, A. G. Hayes, L. M. Prockter, B. P. Weiss, C. A. Hibbitts, A. Moussessian, T. G. Brockwell, H.-W. Hsu, X. Jia, G. R. Gladstone, A. S. McEwen, G. W. Patterson, R. L. McNutt, J. P. Evans, T. W. Larson, L. A. Cangahuala, G. G. Havens, B. B. Buffington, B. Bradley, S. Campagnola, S. H. Hardman, J. M. Srinivasan, K. L. Short, T. C. Jedrey, J. A. St. Vaughn, K. P. Clark, J. Vertesi, C. Niebur, Science overview of the Europa Clipper mission. *Space Sci. Rev.* **220**, 40 (2024).

9. J. W. Barnes, E. P. Turtle, M. G. Trainer, R. D. Lorenz, S. M. MacKenzie, W. B. Brinckerhoff, M. L. Cable, C. M. Ernst, C. Freissinet, K. P. Hand, A. G. Hayes, S. M. Hörst, J. R. Johnson, E. Karkoschka, D. J. Lawrence, A. Le Gall, J. M. Lora, C. P. McKay, R. S. Miller, S. L. Murchie, C. D. Neish, C. E. Newman, J. Núñez, M. P. Panning, A. M. Parsons, P. N. Peplowski, L. C. Quick, J. Radebaugh, S. C. R. Rafkin, H. Shiraishi, J. M. Soderblom, K. S. Sotzen, A. M. Stickle, E. R. Stofan, C. Szopa, T. Tokano, T. Wagner, C. Wilson, R. A. Yingst, K. Zacny, S. C. Stähler, Science goals and objectives for the Dragonfly Titan rotorcraft relocatable lander. *Planet. Sci. J.* **2**, 130 (2021).

10. M. Blanc, C. Wang, L. Li, M. Li, L. Wang, Y. Wang, Y. Wang, Q. Zong, N. Andre, O. Mousis, D. Hestroffer, P. Vernazza, "Gan De: Science objectives and mission scenarios for China's mission to the Jupiter system," in *Proceedings of the EGU General Assembly Conference Abstracts* (Copernicus Publications, 2020).

11. J. P. Gardner, J. C. Mather, M. Clampin, R. Doyon, M. A. Greenhouse, H. B. Hammel, J. B. Hutchings, P. Jakobsen, S. J. Lilly, K. S. Long, J. I. Lunine, M. J. McCaughrean, M. Mountain,

J. Nella, G. H. Rieke, M. J. Rieke, H. W. Rix, E. P. Smith, G. Sonneborn, M. Stiavelli, H. S. Stockman, R. A. Windhorst, G. S. Wright, The James Webb space telescope. *Space Sci. Rev.* **123**, 485–606 (2006).

12. C. S. Cockell, T. Bush, C. Bryce, S. Direito, M. Fox-Powell, J. P. Harrison, H. Lammer, H. Landenmark, J. Martin-Torres, N. Nicholson, L. Noack, J. O'Malley-James, S. J. Payler, A. Rushby, T. Samuels, P. Schwendner, J. Wadsworth, M. P. Zorzano, Habitability: A review. *Astrobiology* **16**, 89–117 (2016).

13. A. S. Burton, J. C. Stern, J. E. Elsila, D. P. Glavin, J. P. Dworkin, Understanding prebiotic chemistry through the analysis of extraterrestrial amino acids and nucleobases in meteorites. *Chem. Soc. Rev.* **41**, 5459–5472 (2012).

14. S. Pizzarello, E. Shock, The organic composition of carbonaceous meteorites: The evolutionary story ahead of biochemistry. *Cold Spring Harb. Perspect. Biol.* **2**, a002113 (2010).

15. J. P. Amend, E. L. Shock, Energetics of amino acid synthesis in hydrothermal ecosystems. *Science* **281**, 1659–1662 (1998).

16. B. Ménez, C. Pisapia, M. Andreani, F. Jamme, Q. P. Vanbellingen, A. Brunelle, L. Richard, P. Dumas, M. Réfrégiers, Abiotic synthesis of amino acids in the recesses of the oceanic lithosphere. *Nature*, **564**, 59–63 (2018).

17. L. M. Barge, E. Flores, M. M. Baum, D. G. V. Velde, M. J. Russell, Redox and pH gradients drive amino acid synthesis in iron oxyhydroxide mineral systems. *Proc. Natl. Acad. Sci. U.S.A.* **116**, 4828–4833 (2019).

18. N. Sleep, D. Bird, E. Pope, Serpentinite and the dawn of life. *Philos. Trans. R. Soc. London, Ser. B* **366**, 2857–2869 (2011).

19. X. Zang, Y. Ueno, N. Kitadai, Photochemical synthesis of ammonia and amino acids from nitrous oxide. *Astrobiology* **22**, 387–398 (2022).

20. S. L. Miller, A production of amino acids under possible primitive earth conditions. *Science* **117**, 528–529 (1953).

21. F. Postberg, N. Khawaja, B. Abel, G. Choblet, C. R. Glein, M. S. Gudipati, B. L. Henderson, H. W. Hsu, S. Kempf, F. Klenner, G. Moragas-Klostermeyer, B. Magee, L. Nölle, M. Perry, R. Reviol, J. Schmidt, R. Srama, F. Stolz, G. Tobie, M. Trieloff, J. H. Waite, Macromolecular organic compounds from the depths of Enceladus. *Nature* **558**, 564–568 (2018).

22. K. E. Mandt, D. A. Gell, M. Perry, J. H. Waite Jr, F. A. Crary, D. Young, B. A. Magee, J. H. Westlake, T. Cravens, W. Kasprzak, G. Miller, J.-E. Wahlund, K. Ågren, N. J. T. Edberg, A. N. Heays, B. R. Lewis, S. T. Gibson, V. de la Haye, M.-C. Liang, Ion densities and composition of Titan's upper atmosphere derived from the Cassini Ion Neutral Mass Spectrometer: Analysis methods and comparison of measured ion densities to photochemical model simulations. *J. Geophys. Res. Planets* **117**, E10006 (2012).

23. B. Journaux, K. Kalousová, C. Sotin, G. Tobie, S. Vance, J. Saur, O. Bollengier, L. Noack, T. Rückriemen-Bez, T. Van Hoolst, K. M. Soderlund, J. M. Brown, Large ocean worlds with high-pressure ices. *Space Sci. Rev.* **216**, 7 (2020).

24. K. P. Hand, "Halogens on and within the ocean worlds of the outer solar system" in *The Role of Halogens in Terrestrial and Extraterrestrial Geochemical Processes: Surface, Crust, and Mantle*, D. E. Harlov, L. Aranovich, Eds. (Springer International Publishing, 2018), pp. 997–1016.

25. G. Choblet, G. Tobie, C. Sotin, K. Kalousová, O. Grasset, Heat transport in the high-pressure ice mantle of large icy moons. *Icarus* **285**, 252–262 (2017).

26. K. Kalousová, C. Sotin, G. Choblet, G. Tobie, O. Grasset, Two-phase convection in Ganymede's high-pressure ice layer—Implications for its geological evolution. *Icarus* **299**, 133–147 (2018).

27. G. W. Gross, R. K. Svec, Effect of ammonium on anion uptake and dielectric relaxation in laboratory-grown ice columns. *Journal of Physical Chemistry B* **101**, 6282–6284 (1997).

28. L. E. Bove, R. Gaal, Z. Raza, A. A. Ludl, S. Klotz, A. M. Saitta, A. F. Goncharov, P. Gillet, Effect of salt on the H-bond symmetrization in ice. *Proc. Natl. Acad. Sci. U.S.A.* **112**, 8216–8220 (2015).

29. M. R. Frank, E. Aarestad, H. P. Scott, V. B. Prakapenka, A comparison of ice VII formed in the H_2O, NaCl-H_2O, and CH_3OH-H_2O systems: Implications for H_2O-rich planets. *Phys. Earth Planet. Inter.* **215**, 12–20 (2013).

30. M. R. Frank, H. P. Scott, E. Aarestad, V. B. Prakapenka, Potassium chloride-bearing ice VII and ice planet dynamics. *Geochim. Cosmochim. Acta* **174**, 156–166 (2016).

31. M. R. Frank, H. P. Scott, S. J. Maglio, V. B. Prakapenka, G. Shen, Temperature induced immiscibility in the NaCl-H_2O system at high pressure. *Phys. Earth Planet. Inter.* **170**, 107–114 (2008).

32. M. R. Frank, C. E. Runge, H. P. Scott, S. J. Maglio, J. Olson, V. B. Prakapenka, G. Shen, Experimental study of the NaCl-H_2O system up to 28 GPa: Implications for ice-rich planetary bodies. *Phys. Earth Planet. Inter.* **155**, 152–162 (2006).

33. S. Klotz, K. Komatsu, F. Pietrucci, H. Kagi, A. A. Ludl, S. Machida, T. Hattori, A. Sano-Furukawa, L. E. Bove, Ice VII from aqueous salt solutions: From a glass to a crystal with broken H-bonds. *Sci. Rep.* **6**, 32040 (2016).

34. J. A. Hernandez, R. Caracas, S. Labrosse, Stability of high-temperature salty ice suggests electrolyte permeability in water-rich exoplanet icy mantles. *Nat. Commun.* **13**, 3303 (2022).

35. C. G. Salzmann, Z. Sharif, C. L. Bull, S. T. Bramwell, A. Rosu-Finsen, N. P. Funnell, Ammonium fluoride as a hydrogen-disordering agent for ice. *J. Phys. Chem. C* **123**, 16486–16492 (2019).

36. B. Journaux, I. Daniel, S. Petitgirard, H. Cardon, J.-P. Perrillat, R. Caracas, M. Mezouar, Salt partitioning between water and high-pressure ices. Implication for the dynamics and habitability of icy moons and water-rich planetary bodies. *Earth Planet. Sci. Lett.* **463**, 36–47 (2017).

37. S. Klotz, L. E. Bove, T. Strässle, T. C. Hansen, A. M. Saitta, The preparation and structure of salty ice VII under pressure. *Nat. Mater.* **8**, 405–409 (2009).

38. B. Journaux, Salty ice and the dilemma of ocean exoplanet habitability. *Nat. Commun.* **13**, 3304 (2022).

39. J. C. Aponte, J. E. Elsila, J. E. Hein, J. P. Dworkin, D. P. Glavin, H. L. McLain, E. T. Parker, T. Cao, E. L. Berger, A. S. Burton, Analysis of amino acids, hydroxy acids, and amines in CR chondrites. *Meteorit. Planet. Sci.* **55**, 2422–2439 (2020).

40. J. Kapitán, V. Baumruk, V. Kopecký Jr, P. Bouř, Conformational flexibility of L-alanine zwitterion determines shapes of Raman and Raman optical activity spectral bands. *J. Phys. Chem.* **110**, 4689–4696 (2006).

41. B. Journaux, I. Daniel, R. Caracas, G. Montagnac, H. Cardon, Influence of NaCl on ice VI and ice VII melting curves up to 6GPa, implications for large icy moons. *Icarus* **226**, 355–363 (2013).

42. Y. Zhang, N. Wei, L. Li, Y. Liu, C. Huang, Z. Li, Y. Huang, D. Zhang, J. S. Francisco, J. Zhao, C. Wang, X. C. Zeng, Fully atomistic molecular dynamics simulation of ice nucleation near an antifreeze protein. *J. Am. Chem. Soc.* **147**, 4411–4418 (2025).

43. A. K. Balcerzak, M. Febbraro, R. N. Ben, The importance of hydrophobic moieties in ice recrystallization inhibitors. *RSC Adv.* **3**, 3232–3236 (2013).

44. S. Devarakonda, J. M. B. Evans, A. Y. Lee, A. S. Myerson, Molecular dynamics study of the interactions of ice inhibitors on the ice {001} surface. *Langmuir* **20**, 5353–5357 (2004).

45. M. Meot-Ner, The ionic hydrogen bond. *Chem. Rev.* **105**, 213–284 (2005).

46. M. G. Campo, Molecular dynamics simulation of glycine zwitterion in aqueous solution. *J. Chem. Phys.* **125**, 114511 (2006).

47. M. Di Gioacchino, M. A. Ricci, S. Imberti, N. Holzmann, F. Bruni, Hydration and aggregation of a simple amino acid: The case of glycine. *J. Mol. Liq.* **301**, 112407 (2020).

48. M. Benoit, D. Marx, M. Parrinello, Tunnelling and zero-point motion in high-pressure ice. *Nature* **392**, 258–261 (1998).

49. B. Cheng, E. A. Engel, J. Behler, C. Dellago, M. Ceriotti, Ab initio thermodynamics of liquid and solid water. *Proc. Natl. Acad. Sci. U.S.A.* **116**, 1110–1115 (2019).

50. C. P. Herrero, R. Ramírez, Path-integral simulation of ice VII: Pressure and temperature effects. *Chem. Phys.* **461**, 125–136 (2015).

51. T. E. Markland, M. Ceriotti, Nuclear quantum effects enter the mainstream. *Nat. Rev. Chem.* **2**, 0109 (2018).

52. T. Meier, S. Petitgirard, S. Khandarkhaeva, L. Dubrovinsky, Observation of nuclear quantum effects and hydrogen bond symmetrisation in high pressure ice. *Nat. Commun.* **9**, 2766 (2018).

53. J. Tsuchiya, M. Shiga, S. Tsuneyuki, E. C. Thompson, Nuclear quantum effect on the elasticity of ice VII under pressure: A path-integral molecular dynamics study. *Phys. Rev. Res.* **6**, 023302 (2024).

54. J.-A. Hernandez, R. Caracas, Proton dynamics and the phase diagram of dense water ice. *J. Chem. Phys.* **148**, 214501 (2018).

55. T. Grützner, H. Bureau, Heavy halogen impact on Raman water bands at high pressure: Implications for salinity estimations in fluid inclusions. *Chem. Geol.* **654**, 122065 (2024).

56. W. P. Hsieh, Y. H. Chien, High pressure Raman spectroscopy of H_2O-CH_3OH mixtures. *Sci. Rep.* **5**, 8532 (2014).

57. J. Hao, E. Giovenco, U. Pedreira-Segade, G. Montagnac, I. Daniel, Compatibility of amino acids in ice Ih: Implications for the origin of life. *Astrobiology* **18**, 381–392 (2018).

58. C. A. Knight, A. Wierzbicki, Adsorption of biomolecules to ice and their effects upon ice growth. 2. A discussion of the basic mechanism of “antifreeze” phenomena. *Cryst. Growth Des.* **1**, 439–446 (2001).

59. G. Tzvetkov, M. G. Ramsey, F. P. Netzer, Glycine-ice nanolayers: Morphology and surface energetics. *J. Chem. Phys.* **122**, 114712 (2005).

60. J.-H. Sa, G.-H. Kwak, K. Han, D. Ahn, S. J. Cho, J. D. Lee, K.-H. Lee, Inhibition of methane and natural gas hydrate formation by altering the structure of water with amino acids. *Sci. Rep.* **6**, 31582 (2016).

61. M. Choukroun, Y. Morizet, O. Grasset, Raman study of methane clathrate hydrates under pressure: New evidence for the metastability of structure II. *J. Raman Spectrosc.* **38**, 440–451 (2007).

62. B. Li, Y.-Y. Lu, Y.-L. Li, A review of natural gas hydrate formation with amino acids. *J. Mar. Sci. Eng.* **10**, 1134 (2022).

63. J.-H. Sa, B. R. Lee, D.-H. Park, K. Han, H. D. Chun, K.-H. Lee, Amino acids as natural inhibitors for hydrate formation in CO_2 sequestration. *Environ. Sci. Technol.* **45**, 5885–5891 (2011).

64. E. D. Sloan Jr., C. A. Koh, *Clathrate Hydrates of Natural Gases* (CRC Press, 2007).

65. H. P. Veluswamy, R. Kumar, P. Linga, Hydrogen storage in clathrate hydrates: Current state of the art and future directions. *Appl. Energy* **122**, 112–132 (2014).

66. A. D. Fortes, M. Choukroun, Phase behaviour of ices and hydrates. *Space Sci. Rev.* **153**, 185–218 (2010).

67. J.-H. Sa, G.-H. Kwak, B. R. Lee, D. Ahn, K.-H. Lee, Abnormal incorporation of amino acids into the gas hydrate crystal lattice. *Phys. Chem. Chem. Phys.* **16**, 26730–26734 (2014).

68. C. Holzammer, A. Finckenstein, S. Will, A. S. Braeuer, How sodium chloride salt inhibits the formation of CO_2 gas hydrates. *J. Phys. Chem. B* **120**, 2452–2459 (2016).

69. U. Pedreira-Segade, J. Hao, G. Montagnac, H. Cardon, I. Daniel, Spontaneous polymerization of glycine under hydrothermal conditions. *ACS Earth Space Chem.* **3**, 1669–1677 (2019).

70. J. S. Cox, T. M. Seward, The reaction kinetics of alanine and glycine under hydrothermal conditions. *Geochim. Cosmochim. Acta* **71**, 2264–2284 (2007).

71. N. Truong, A. A. Monroe, C. R. Glein, A. D. Anbar, J. I. Lunine, Decomposition of amino acids in water with application to in-situ measurements of Enceladus, Europa and other hydrothermally active icy ocean worlds. *Icarus* **329**, 140–147 (2019).

72. T. Otake, T. Taniguchi, Y. Furukawa, F. Kawamura, H. Nakazawa, T. Kakegawa, Stability of amino acids and their oligomerization under high-pressure conditions: Implications for prebiotic chemistry. *Astrobiology* **11**, 799–813 (2011).

73. Á. Boldog, V. Dobos, L. L. Kiss, M. Van Der Perk, A. C. Barr, Water content of rocky exoplanets in the habitable zone. *Astron. Astrophys.* **681** (2024), A109.

74. A. Néri, F. Guyot, B. Reynard, C. Sotin, A carbonaceous chondrite and cometary origin for icy moons of Jupiter and Saturn. *Earth Planet. Sci. Lett.* **530**, 115920 (2020).

75. B. Reynard, C. Sotin, Carbon-rich icy moons and dwarf planets. *Earth Planet. Sci. Lett.* **612**, 118172 (2023).

76. E. L. Steel, A. Davila, C. P. Mckay, Abiotic and biotic formation of amino acids in the Enceladus ocean. *Astrobiology* **17**, 862–875 (2017).

77. E. Shock, P. Canovas, The potential for abiotic organic synthesis and biosynthesis at seafloor hydrothermal systems. *Geofluids*, **10**, 161, 192 (2010).

78. M. Andreani, G. Montagnac, C. Fellah, J. Hao, F. Vandier, I. Daniel, C. Pisapia, J. Galipaud, M. D. Lilley, G. L. Früh Green, S. Borensztajn, B. Ménez, The rocky road to organics needs drying. *Nat. Commun.* **14**, 347 (2023).

79. L. Lebec, S. Labrosse, A. Morison, P. J. Tackley, Scaling of convection in high-pressure ice layers of large icy moons and implications for habitability. *Icarus* **396**, 115494 (2023).

80. K. Kalousová, C. Sotin, Melting in high-pressure ice layers of large ocean worlds—Implications for volatiles transport. *Geophys. Res. Lett.* **45**, 8096–8103 (2018).

81. K. Kalousová, C. Sotin, Dynamics of Titan's high-pressure ice layer. *Earth Planet. Sci. Lett.* **545**, 116416 (2020).

82. R. Fu, R. J. O'Connell, D. D. Sasselov, The interior dynamics of water planets. *Astrophys. J.* **708**, 1326–1334 (2010).

83. A. Sharma, J. H. Scott, G. D. Cody, M. L. Fogel, R. M. Hazen, R. J. Hemley, W. T. Huntress, Microbial activity at gigapascal pressures. *Science* **295**, 1514–1516 (2002).

84. N. Madhusudhan, A. A. A. Piette, S. Constantinou, Habitability and biosignatures of hycean worlds. *Astrophys. J.* **918**, 1 (2021).

85. J. E. Elsila, D. P. Glavin, J. P. Dworkin, Cometary glycine detected in samples returned by Stardust. *Meteorit. Planet. Sci.* **44**, 1323–1330 (2009).

86. D. P. Glavin, J. P. Dworkin, S. A. Sandford, Detection of cometary amines in samples returned by Stardust. *Meteorit. Planet. Sci.* **43**, 399–413 (2008).

87. K. Altwegg, H. Balsiger, A. Bar-Nun, J.-J. Berthelier, A. Bieler, P. Bochsler, C. Briois, U. Calmonte, M. R. Combi, H. Cottin, J. De Keyser, F. Dhooghe, B. Fiethe, S. A. Fuselier, S. Gasc, T. I. Gombosi, K. C. Hansen, M. Haessig, A. Jäckel, E. Kopp, A. Korth, L. Le Roy, U. Mall, B. Marty, O. Mousis, T. Owen, H. Rème, M. Rubin, T. Sémon, C.-Y. Tzou, J. H. Waite, P. Wurz, Prebiotic chemicals—amino acid and phosphorus—in the coma of comet 67P/Churyumov-Gerasimenko. *Sci. Adv.* **2**, e1600285 (2016).

88. K. Hadraoui, H. Cottin, S. L. Ivanovski, P. Zapf, K. Altwegg, Y. Benilan, N. Biver, V. Della Corte, N. Fray, J. Lasue, S. Merouane, A. Rotundi, V. Zakharov, Distributed glycine in comet 67P/Churyumov-Gerasimenko. *Astron. Astrophys.* **630**, A32 (2019).

89. W. R. M. Rocha, E. F. van Dishoeck, M. E. Ressler, M. L. van Gelder, K. Slavicinska, N. G. C. Brunken, H. Linnartz, T. P. Ray, H. Beuther, A. Caratti o Garatti, V. Geers, P. J. Kavanagh, P. D. Klaassen, K. Justtanont, Y. Chen, L. Francis, C. Gieser, G. Perotti, Ł. Tychoniec, M. Barsony, L. Majumdar, V. J. M. le Gouellec, L. E. U. Chu, B. W. P. Lew, T. Henning, G. Wright, JWST Observations of Young protoStars (JOYS+): Detecting icy complex organic molecules and ions: I. CH_4, SO_2, HCOO, OCN, H_2CO, HCOOH, CH_3CH_2OH, CH_3CHO, CH_3OCHO, and CH_3COOH. *Astron. Astrophys.* **683**, A124 (2024).

90. E. T. Parker, Q. H. S. Chan, D. P. Glavin, J. P. Dworkin, Non-protein amino acids identified in carbon-rich Hayabusa particles. *Meteorit. Planet. Sci.* **57**, 776–793 (2022).

91. D. P. Glavin, J. P. Dworkin, C. M. O'D Alexander, J. C. Aponte, A. A. Baczynski, J. J. Barnes, H. A. Bechtel, E. L. Berger, A. S. Burton, P. Caselli, A. H. Chung, S. J. Clemett, G. D. Cody, G. Dominguez, J. E. Elsila, K. K. Farnsworth, D. I. Foustoukos, K. H. Freeman, Y. Furukawa, Z. Gainsforth, H. V. Graham, T. Grassi, B. M. Giuliano, V. E. Hamilton, P. Haenecour, P. R. Heck, A. E. Hofmann, C. H. House, Y. Huang, H. H. Kaplan, L. P. Keller, B. Kim, T. Koga, M. Liss, H. L. McLain, M. A. Marcus, M. Matney, T. J. McCoy, O. M. McIntosh, A. Mojarro, H. Naraoka, A. N. Nguyen, M. Nuevo, J. A. Nuth III, Y. Oba, E. T. Parker, T. S. Peretyazhko, S. A. Sandford, E. Santos, P. Schmitt-Kopplin, F. Seguin, D. N. Simkus, A. Shahid, Y. Takano, K. L. Thomas-Keprta, H. Tripathi, G. Weiss, Y. Zheng, N. G. Lunning, K. Righter, H. C. Connolly Jr, D. S. Lauretta, Abundant ammonia and nitrogen-rich soluble organic matter in samples from asteroid (101955) Bennu. *Nat. Astron.* **9**, 199–210 (2025).

92. N. Goldman, E. J. Reed, L. E. Fried, I. F. William Kuo, A. Maiti, Synthesis of glycine-containing complexes in impacts of comets on early Earth. *Nat. Chem.* **2**, 949–954 (2010).

93. Z. Martins, M. C. Price, N. Goldman, M. A. Sephton, M. J. Burchell, Shock synthesis of amino acids from impacting cometary and icy planet surface analogues. *Nature Geosci* **6**, 1045–1049 (2013).

94. M. A. Leitner, J. I. Lunine, Modeling early Titan's ocean composition. *Icarus* **333**, 61–70 (2019).

95. M. Y. Zolotov, Aqueous fluid composition in CI chondritic materials: Chemical equilibrium assessments in closed systems. *Icarus* **220**, 713–729 (2012).

96. K. J. Robinson, C. Bockisch, I. R. Gould, Y. Liao, Z. Yang, C. R. Glein, G. D. Shaver, H. E. Hartnett, L. B. Williams, E. L. Shock, Quantifying the extent of amide and peptide bond synthesis across conditions relevant to geologic and planetary environments. *Geochim. Cosmochim. Acta* **300**, 318–332 (2021).

97. J. C. Chervin, B. Canny, J. M. Besson, P. Pruzan, A diamond anvil cell for IR microspectroscopy. *Rev. Sci. Instrum.* **66**, 2595–2598 (1995).

98. J. C. Chervin, B. Canny, M. Mancinelli, Ruby-spheres as pressure gauge for optically transparent high pressure cells. *High Pressure Res.* **21**, 305–314 (2001).

99. H. K. Mao, J. Xu, P. M. Bell, Calibration of the ruby pressure gauge to 800 kbar under quasi-hydrostatic conditions. *J. Geophys. Res. Solid Earth* **91**, 4673–4676 (1986).

100. G. J. Piermarini, S. Block, J. D. Barnett, R. A. Forman, Calibration of the pressure dependence of the R1 ruby fluorescence line to 195 kbar. *J. Appl. Phys.* **46**, 2774–2780 (1975).

101. S. Roy, A. Hossain, B. K. Dolui, Solubility and chemical thermodynamics of d,l-alanine and d,l-serine in aqueous NaCl and KCl solutions. *J. Chem. Eng. Data* **61**, 132–141 (2016).

102. P. J. Aarnoutse, J. A. Westerhuis, Quantitative raman reaction monitoring using the solvent as internal standard. *Anal. Chem.* **77**, 1228–1236 (2005).

103. Y. Numata, M. Otsuka, K. Yamagishi, H. Tanaka, Quantitative determination of glycine, alanine, aspartic acid, glutamic acid, phenylalanine, and tryptophan by Raman spectroscopy. *Anal. Lett.* **50**, 651–662 (2017).

104. A. Picard, I. Daniel, G. Montagnac, P. Oger, In situ monitoring by quantitative Raman spectroscopy of alcoholic fermentation by *Saccharomyces cerevisiae* under high pressure. *Extremophiles* **11**, 445–452 (2007).

105. V. Thomsen, D. Schatzlein, D. Mercuro, Limits of detection in spectroscopy: A diagrammatic view of the concept of detection limits is presented that effectively shows its relationship to the reproducibility of measurements on the blank. *Spectroscopy* **18**, 112–115 (2003).

106. F. R. Tortonda, J. L. Pascual-Ahuir, E. Silla, I. Tuñón, F. J. Ramírez, Aminoacid zwitterions in solution: Geometric, energetic, and vibrational analysis using density functional theory-continuum model calculations. *J. Chem. Phys.* **109**, 592–603 (1998).

107. B. Z. Chowdhry, T. J. Dines, S. Jabeen, R. Withnall, Vibrational spectra of α-amino acids in the zwitterionic state in aqueous solution and the solid state: DFT calculations and the influence of hydrogen bonding. *J. Phys. Chem. A* **112**, 10333–10347 (2008).

108. G. Kresse, J. Furthmüller, Efficiency of ab-initio total energy calculations for metals and semiconductors using a plane-wave basis set. *Comput. Mater. Sci.* **6**, 15–50 (1996).

109. J. P. Perdew, K. Burke, M. Ernzerhof, Generalized gradient approximation made simple. *Phys. Rev. Lett.* **77**, 3865–3868 (1996).

110. P. E. Blöchl, Projector augmented-wave method. *Phys. Rev. B* **50**, 17953–17979 (1994).

111. S. Grimme, S. Ehrlich, L. Goerigk, Effect of the damping function in dispersion corrected density functional theory. *J. Comput. Chem.* **32**, 1456–1465 (2011).

112. M. E. Tuckerman, *Statistical Mechanics: Theory and Molecular Simulation* (Oxford University Press, 2023).

113. W. F. Kuhs, J. L. Finney, C. Vettier, D. V Bliss, Structure and hydrogen ordering in ices VI, VII, and VIII by neutron powder diffraction. *J. Chem. Phys.* **81**, 3612–3623 (1984).

114. B. Kamb, B. L. Davis, Ice VII, the densest form of ice. *Proc. Natl. Acad. Sci. U.S.A.* **52**, 1433–1439 (1964).

115. J. L. Kuo, W. F. Kuhs, A first principles study on the structure of Ice-VI: Static distortion, molecular geometry, and proton ordering. *J. Phys. Chem. B* **110**, 3697–3703 (2006).

116. J. L. Kuo, M. L. Klein, Structure of Ice-VII and Ice-VIII: A quantum mechanical study. *J. Phys. Chem. B* **108**, 19634–19639 (2004).

117. J. G. Kirkwood, V. A. Lewinson, B. J. Alder, Radial distribution functions and the equation of state of fluids composed of molecules interacting according to the Lennard-Jones potential. *J. Chem. Phys.* **20**, 929–938 (1952).

118. R. L. Lundblad, F. M. Macdonald, *Handbook of Biochemistry and Molecular Biology* (CRC Press, 2018).

119. H. Matsuo, Y. Suzuki, S. Sawamura, Solubility of α-amino acids in water under high pressure: Glycine, l-alanine, l-valine, l-leucine, and l-isoleucine. *Fluid Phase Equilib.* **200**, 227–237 (2002).

120. S. Shigeto, D. D. Dlott, Vibrational relaxation of an amino acid in aqueous solution. *Chem. Phys. Lett.* **447**, 134–139 (2007).

121. S. Kumar, A. Kumar Rai, S. B. Rai, D. K. Rai, A. N. Singh, V. B. Singh, Infrared, Raman and electronic spectra of alanine: A comparison with ab intio calculation. *J. Mol. Struct.* **791**, 23–29 (2006).
